# Stereo/regio-selective access to substituted 3-hydroxy-oxindoles with anti-proliferative assessment and *in silico* validation†

**DOI:** 10.1039/d3ra05869g

**Published:** 2023-09-27

**Authors:** Preeti, Asif Raza, Amit Anand, Natacha Henry, Arun K. Sharma, Pascal Roussel, Vipan Kumar

**Affiliations:** a Department of Chemistry, Guru Nanak Dev University Amritsar India vipan_org@yahoo.com; b Department of Pharmacology, Penn State Cancer Institute, The Pennsylvania State University College of Medicine Hershey PA 17033 USA; c Department of Chemistry, Khalsa College Amritsar India; d Univ. Lille, CNRS, Centrale Lille, Univ. Artois, UMR 8181, Unité de Catalyse et Chimie du Solide (UCCS) F-59000 Lille France

## Abstract

The manuscript focuses on a highly stereo-/regioselective approach for synthesizing a diverse array of substituted-3-hydroxy-2-oxindoles. The synthesized compounds were subsequently subjected to anti-proliferative assessment against various cell lines, including colorectal carcinoma, ovarian cancer, and human metastatic melanoma cancer. The structural characterization of the synthesized scaffolds was unambiguously confirmed using X-ray diffraction analysis. Among the synthesized compounds, one compound demonstrated exceptional potency within the series. It exhibited 1.2, 2.12, and 1.55 times greater potency than cisplatin against the HCT116, OVCAR10, and 1205Lu cell lines, respectively. These results were further supported by *in vitro* caspase-mediated apoptosis studies. Molecular docking studies of these compounds on the target VEGFR2 protein revealed their binding capability.

## Abstract 2

Anti-proliferative evaluation and *in silico* validation of synthesized substituted-3-hydroxy-2-oxindoles.

## Graphical abstract

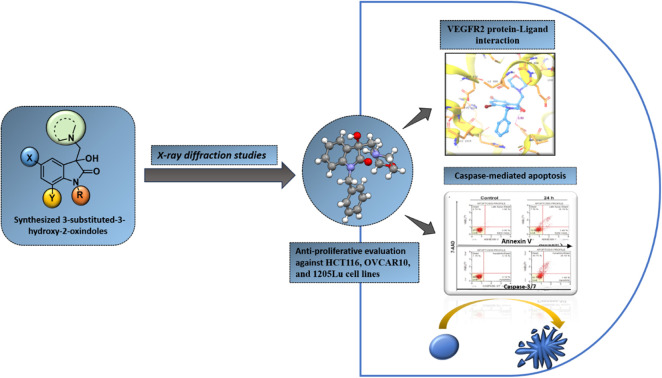

## Introduction

Cancer is the second most common cause of mortality accounting for one in every six fatalities and is challenging to treat. According to data from the American Cancer Society, cancer was predicted to be the cause of nearly 609 820 US fatalities in 2023 which is about 1670 deaths per day.^1^ Vascular endothelial growth factor receptor 2 (VEGFR2) has been identified as a key target for anticancer therapy. VEGFR2 is a member of the tyrosine kinase receptor family, and its inhibition prevents angiogenesis by blocking phosphorylation and dimerization.^2,3^ VEGFR2 is crucial for normal physiological conditions, but its deregulation is associated with cancer.^4^ Tumour cells produce VEGF, which activates VEGFR2 and stimulates the growth of new blood vessels, encouraging tumour angiogenesis and cancer spread.^5,6^ Inhibiting VEGFR2 has emerged as a potential strategy to halt cancer growth, and various VEGFR2 inhibitors have undergone clinical testing, with some approved for use. VEGFR2 inhibitors, including sorafenib, sunitinib, pazopanib, lenvatinib, vandetanib, and cabozantinib, have been approved for use by the FDA in treating different types of cancers such as thyroid cancer, renal cell carcinoma, and hepatocellular carcinoma.^7,8^ However, these drugs have limitations such as resistance and side effects, necessitating the identification of newer, more effective scaffolds with fewer side effects.

Isatin (1*H*-indol-2,3-dione) is a naturally occurring heterocyclic compound found in various plants such as *Isatis tinctoria*, *Couroupita guianensis Aubl*, *Melochia tomentosa*, and *Boronia koniamboensis*.^9^ Additionally, it is discovered in the parotid gland of Bufo frogs and *Dicathais orbita*, an Australian mollusc.^10^ It is recognised as a tryptophan or epinephrine metabolite in humans and is found in body fluids, peripheral tissues, and the central nervous system.^11^ Isatin offers a versatile platform for structural modification and derivatization due to its molecular architecture. It can take on the roles of an electrophile and a nucleophile. A variety of isatin derivatives have biological properties that include anti-cancer, anti-fungal, anti-HIV, and anti-inflammatory, *etc.*^12^

**Fig. 1:**
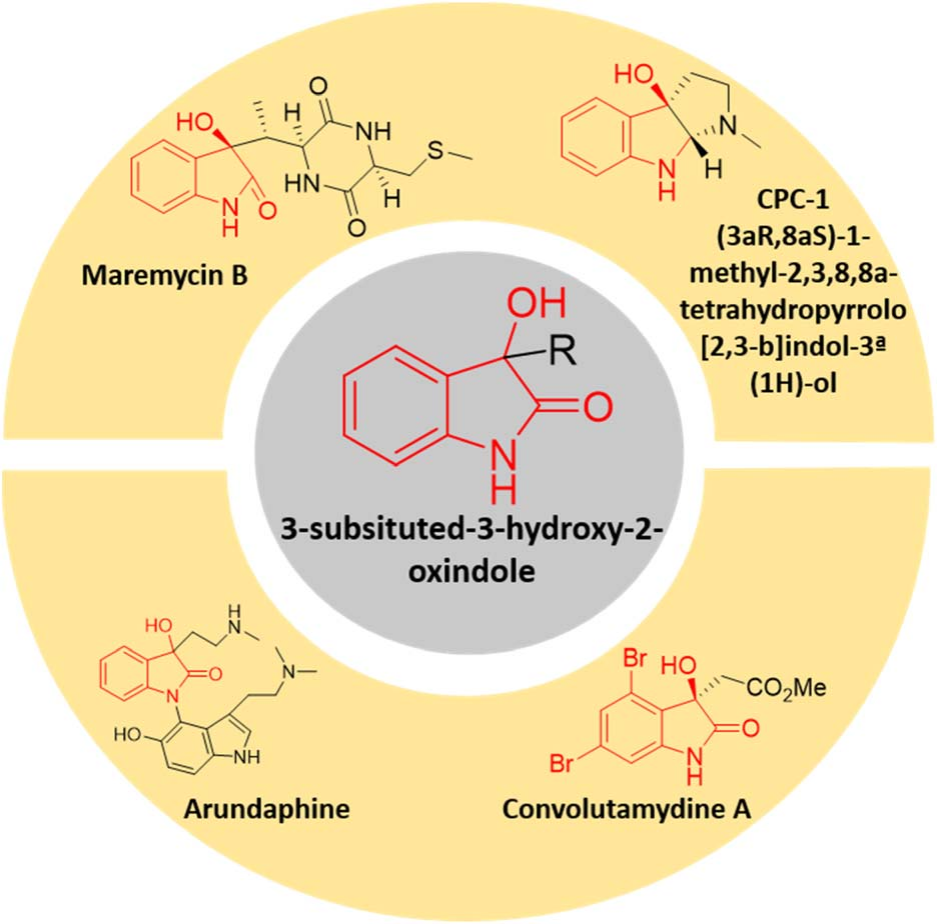
Few examples of bioactive 3-substituted-3-hydroxy-2-oxindole natural products.

In the realm of organic synthesis, heterocyclic fused three-membered ring system has been crucial. The inherent ring strain of these molecules can be harnessed into ring-opening/expansion reactions.^13^ Due to their high reactivity and frequent presence in bioactive small molecules and several natural compounds, epoxides are well-known to both organic and medicinal chemists.^14^ Spiro-epoxyoxindoles possessing an epoxide ring fused to the C-3 position of the oxindole core are regarded as complex building blocks. The presence of heteroatoms along with inherent ring strain makes them susceptible to nucleophilic ring-opening reactions.^15^ The potential utility of these privileged frameworks in organic synthesis as well as drug discovery has lured many synthetic as well as medicinal chemists.^16^ The spiro-epoxyoxindoles have been predominantly known for their nucleophilic ring-opening and Friedel–Crafts arylation reactions to give 3,3-disubstituted oxindoles and spiro-oxindoles.^17^ The 3-substituted-3-hydroxy-2-oxindole scaffold is indeed a significant structural motif found in several natural compounds with diverse biological activities. This scaffold has garnered attention in the field of medicinal chemistry due to the potent properties it exhibits including antioxidative, anticancer, anti-HIV, neuroprotective effects, and other biological characteristics.^18^ There is an expanding list of bioactive 3-substituted-3-hydroxy-2-oxindole natural products, such as convolutamydines, arundaphine, maremycins, CPC-1, *etc.* as shown in Fig. 1. The 3-substituted-3-hydroxy-2-oxindoles have also found their place as targets in the arena of drug discovery in addition to being key intermediates towards complex natural product synthesis.

Our research team recently showed that adding tetrahydro-β-carboline (THβC) to spiro-epoxy oxindoles resulted in 3-THβC-substituted-3-hydroxy-oxindole with good antiproliferative effects against the MCF-7 (ER+) and MDA-MB-231 (ER) cell lines.^19^ Building upon previous studies,^20–22^ this manuscript focuses on the design, synthesis, characterization, and anti-proliferative evaluation of scaffolds that incorporate substituted *N*-benzylated oxindoles with various secondary amines through nucleophilic ring opening of corresponding oxiranes. To compare the effect of the presence of the *N*-benzyl group with the free –NH group of the isatin core, the corresponding unsubstituted oxindole counterparts have also been synthesised and evaluated.

## Results and discussions

### Synthetic chemistry

**Scheme 1:**
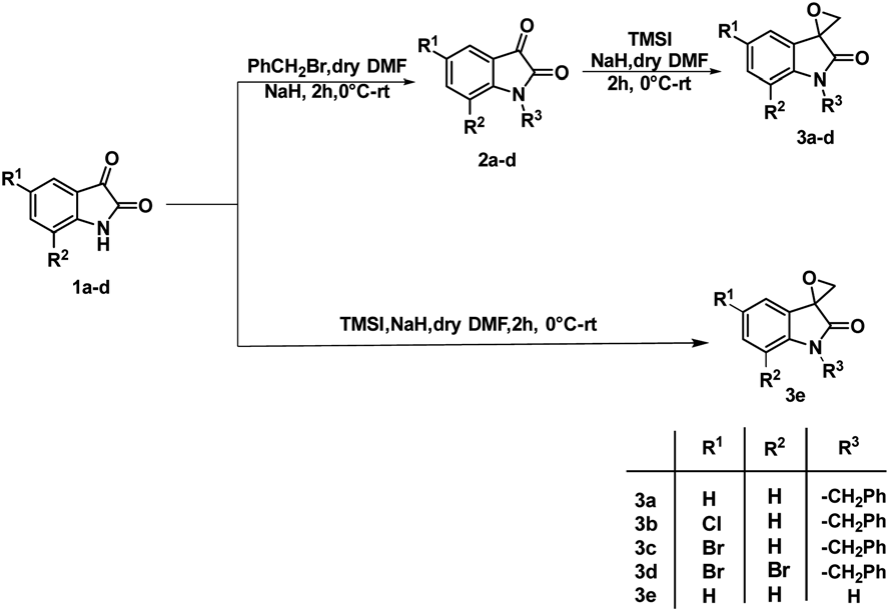
Synthetic route to spiro-epoxyoxindoles 3a–e.

The synthesis of 3-substituted 3-hydroxy-oxindole was initiated with the preparation of a precursor spiro-epoxyoxindole, which was achieved through the Corey–Chaykovsky epoxidation reaction. Spiro-epoxyoxindole 3e was obtained by treating isatin with trimethyl sulfoxonium iodide (TMSI) in the presence of sodium hydride and DMF. For the preparation of *N*-benzylated spiro-epoxyoxindoles 3a–d, a base-promoted benzylation of substituted isatins 1a–d were carried out resulting in the formation of *N*-benzylated isatins 2a–d. The subsequent treatment of 2a–d with trimethyl sulfoxonium iodide (TMSI) yielded the desired *N*-benzylated spiro-epoxyoxindoles 3a–d (Scheme 1).

**Scheme 2:**
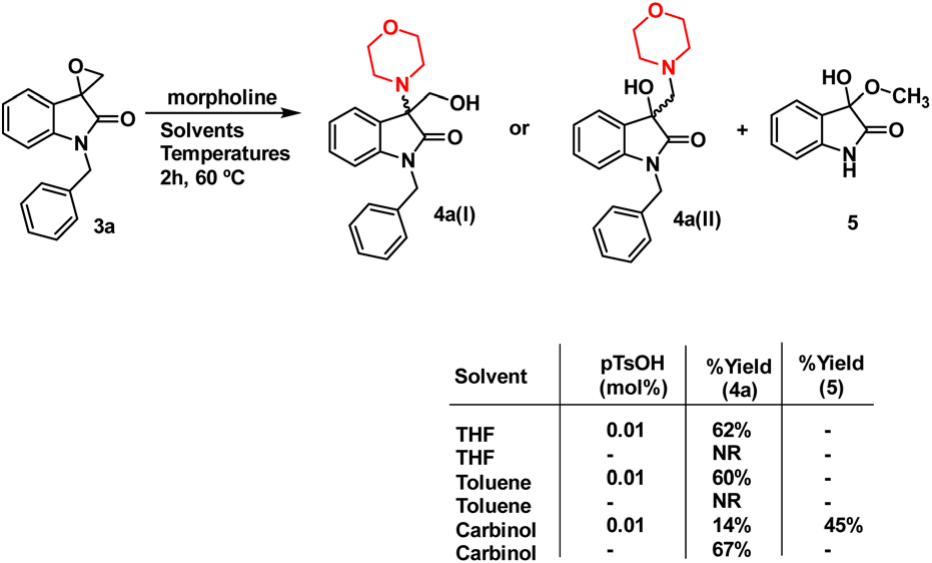
Effect of different solvents and catalytic *p*-TsOH on % yield of 4a and 5.

To optimise the conditions of ring opening, a model reaction between spiro-epoxyoxindole 3a and morpholine was carried out in distinct solvents, namely non-polar toluene, polar aprotic tetrahydrofuran (THF), and polar protic carbinol in the presence or absence of a catalytic quantity of *p*-toluene sulfonic acid (*p*-TsOH) (Scheme 2). As evident, the presence of *p*-TsOH as catalyst promoted the reaction specifically in case of THF and toluene, primarily by coordinating with the oxirane oxygen and facilitating the ring opening. The opening of oxirane ring resulted in the formation of 4a, the structure of which could either be 4a(I), resulted from nucleophilic ring opening from less hindered site or 4b(II) resulted from ring opening from more hindered site. However, in case of carbinol, the presence of catalytic amount of *p*-TsOH led to a mixture of products 4a and 5. Compound 5 was formed by the participation of solvent as the nucleophile.

**Fig. 2:**
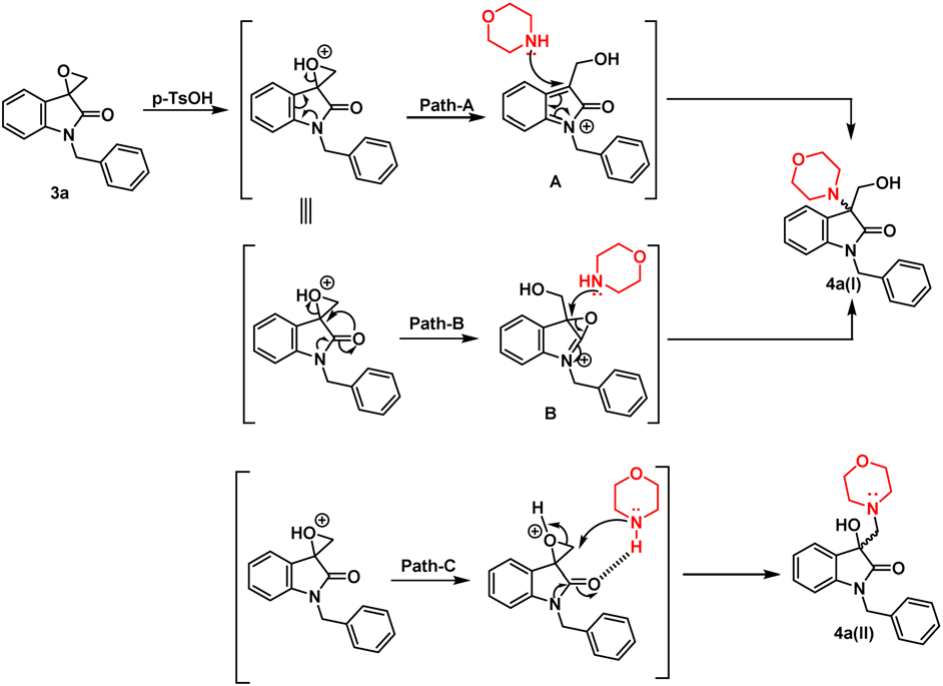
Possible mechanism in the presence of non-polar/polar aprotic solvent in the presence of *p*-TsOH.

In terms of the mechanism, three possible pathways can be proposed for the ring opening in the presence of *p*-TsOH. The initial protonation of the oxirane ring is facilitated by *p*-TsOH. Path A involves the formation of a stable carbocation and subsequent ring opening, followed by the attack of morpholine. On the other hand, path B involves the assistance of cyclic amidic oxygen, leading to ring opening. In both cases, the ring opens at a site that is more hindered, resulting in the formation of a thermodynamically stable product 4a(I).^23,24^ Furthermore, morpholine, being a good nucleophile, can also attack from the less hindered site immediately after protonation, leading to the formation of a product 4a(II) that is controlled by kinetics, according to path C (Fig. 2).

**Fig. 3:**
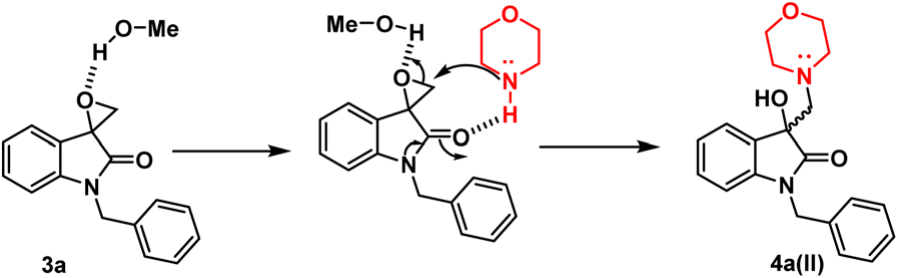
Possible mechanism in presence of polar protic solvent in absence of *p*-TsOH.

However, in the absence of *p*-TsOH, a pseudo S_N_2 mechanism can be proposed for the ring opening, which yields a kinetically controlled product. Initially, the solvent promotes the activation of the epoxide ring. The transition state I (TS-I) is stabilized through hydrogen bonding, and subsequently, with the assistance of cyclic amidic oxygen, nucleophilic ring opening occurs from the less hindered site, resulting in the formation of product 4a(II) (Fig. 3).

**Fig. 4:**
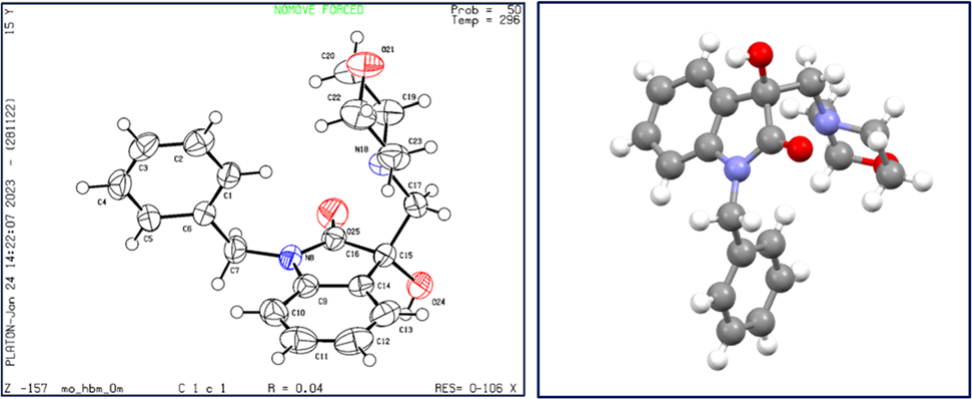
The structure of 4a, showing 50% probability displacement ellipsoids and atomic numbering (left) and 3D structure of crystal lattice (right).

X-ray diffraction studies were performed to unequivocally establish the structure of 4a. Evidently, the epoxide ring opening proceeded *via* nucleophilic attack from the less hindered position as confirmed by the crystal structure, shown in Fig. 4. Table 1 provides statistics on crystallographic data and refinement.

**Table 1:** Crystal data and structure refinement for 4a

Empirical formula	C_20_H_22_N_2_O_3_
Formula weight	338.39
Temperature/K	296
Crystal system	Monoclinic
Space group	*Cc*
*a*/Å	11.5519(5)
*b*/Å	21.2965(11)
*c*/Å	9.1698(5)
*α*/°	90
*β*/°	128.8251(15)
*γ*/°	90
Volume/Å^3^	1757.49(16)
*Z*	4
*ρ*_calc_/g cm^−3^	1.279
*μ*/mm^−1^	0.087
*F*(000)	720.0
Crystal size/mm^3^	0.383 × 0.3 × 0.09
Radiation	MoKα (*λ* = 0.71073)
2*Θ* range for data collection/°	3.824 to 52.764
Index ranges	−14 ≤ *h* ≤ 14, −26 ≤ *k* ≤ 26, −11 ≤ *l* ≤ 11
Reflections collected	7731
Independent reflections	3406 [*R*_int_ = 0.0450, *R*_sigma_ = 0.0586]
Data/restraints/parameters	3406/2/228
Goodness-of-fit on *F*^2^	1.039
Final *R* indexes [*I* ≥ 2*σ*(*I*)]	*R*_1_ = 0.0419, w*R*_2_ = 0.1003
Final *R* indexes [all data]	*R*_1_ = 0.0515, w*R*_2_ = 0.1056
Largest diff. peak/hole/e Å^−3^	0.19/−0.15
Flack parameter	−1.4(10)

Furthermore, analytical evidence and spectral data were used to characterise synthesised molecule 4a. It exhibited a molecular ion peak [M]^+^ at 338.1642 in its High Resolution Mass Spectrum (HRMS). ^1^H NMR of 4a showed characteristic pair of doublets at *δ* 5.07 and 4.72 with a coupling constant of 15.75 corresponding to benzylic –CH_2_ and a multiplet at *δ* 3.67–3.58 corresponding to methylene groups of morpholine –CH_2_-O-CH_2_. The absorption peaks at *δ* 177.46 and 108.35 in the ^13^C NMR spectrum, corresponding to carbonyl and methine groups of isatin further validated the assigned structure.

**Scheme 3:**
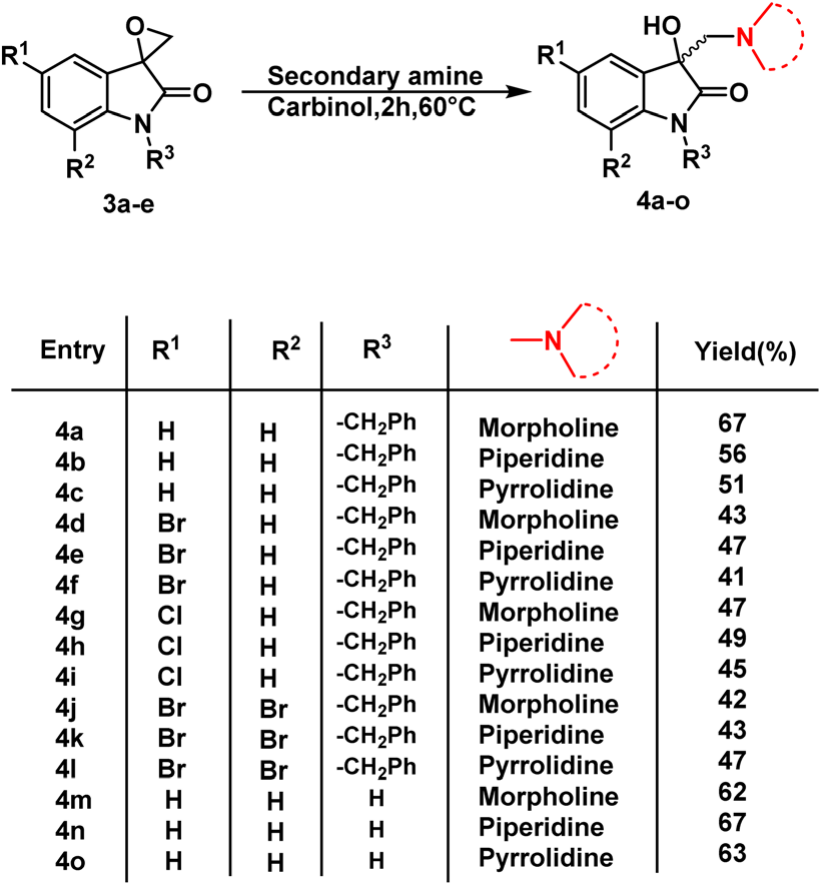
Synthetic route to synthesis of 3-substituted 3-hydroxy-oxindole 4a–o.

Having determined the suitable conditions, the desired scaffolds, 3-substituted 3-hydroxy-oxindoles were synthesized by heating the spiro-epoxyoxindoles with various secondary amines *viz.* morpholine/piperidine/pyrrolindine using carbinol as a solvent for 2 h at 60 °C (Scheme 3). The crude products thus obtained were purified *via* column chromatography using ethyl acetate/hexane (85 : 15) as an eluent.

### *In vitro* studies

#### Biological evaluation and SAR

The newly synthesized compounds were subjected to assessment for their potential to inhibit the proliferation of various cancer cell lines including colorectal carcinoma (HCT116), ovarian cancer (OVCAR 10), and human metastatic melanoma (1205Lu), and non-cancerous fetal human cells (FHC). The MTS assay was employed, and the resulting IC_50_ values are tabulated in Table 2. It is evident that the majority of the synthesized compounds did not demonstrate noteworthy cytotoxic effects against the tested cell lines. However, one compound, 4j, displayed remarkable inhibitory activities across all tested cell lines. Notably, compound 4j exhibited an IC_50_ value of 9.63 μM, rendering it particularly effective against the HCT116 cell line. This efficacy surpassed that of the reference drugs 5-fluorouracil and cisplatin, which showed IC_50_ values of 22.26 μM and 12.11 μM, respectively. Additionally, 4j emerged as the most potent analog in the series against the OVCAR 10 cell line, with an IC_50_ value of 8.56 μM, outperforming cisplatin (IC_50_ = 18.22 μM) and 5-fluorouracil (IC_50_ = 22.4 μM). Furthermore, in comparison to 5-fluorouracil (IC_50_ = 15.4 μM) and cisplatin (IC_50_ = 19.01 μM), compound 4j displayed significant effectiveness against the 1205Lu cell line, exhibiting an IC_50_ value of 12.22 μM. In order to ascertain whether compound 4j is selectively cytotoxic or anti-proliferative to cancer cells, it was tested for non-cancerous fetal human cells (FHC). The compound displayed acceptable cytotoxicity towards FHC with an IC_50_ of 20.56 μM which was comparable to that of standards 5-FU and cisplatin having IC_50_ values of 21.3 and 25.13 μM, respectively.

**Table 2:** The IC_50_(μM) values of the synthesized compounds against HCT116, OVCAR10, 1205Lu, and FHC cell lines

Compound	IC_50_ (μM) ± SD			
	HCT116	OVCAR 10	1205Lu	FHC
4a	104.21 ± 24.96	119.54 ± 15.36	111 ± 18.94	121.51 ± 13.21
4b	85.24 ± 15.26	69.21 ± 16.22	65.27 ± 15.31	63.52 ± 22.23
4c	65.92 ± 16.72	61.25 ± 13.21	78.22 ± 24	76.29 ± 14.51
4d	84.14 ± 19.35	87.88 ± 13.02	81.23 ± 15.1	73.25 ± 19.63
4e	92.20 ± 17.20	93.12 ± 15.62	98 ± 13.2	88.32 ± 16.85
4f	85.32 ± 15.98	88.54 ± 18.88	85.64 ± 13.21	81.26 ± 15.58
4g	121.0 ± 17.12	112.32 ± 11.74	125.26 ± 19.82	121.08 ± 21.25
4h	117.28 ± 19.63	130.39 ± 17.23	110.28 ± 18.36	106.23 ± 17.23
4i	93.42 ± 19.27	81.48 ± 12.54	96.35 ± 18.76	96.52 ± 22.21
4j	9.63 ± 1.3	8.56 ± 1.1	12.22 ± 1.7	20.56 ± 3.1
4k	109.53 ± 24.35	91.23 ± 18.66	89.85 ± 16.83	88.56 ± 20.33
4l	91.48 ± 26.85	94.32 ± 18.02	91.57 ± 18.31	94.75 ± 15.32
4m	95.36 ± 09.66	92.31 ± 18.17	99.65 ± 11.52	89.36 ± 14.57
4n	105.36 ± 23.28	112.33 ± 21.28	109.47 ± 24.74	96.75 ± 18.52
4o	102.03 ± 18.74	116.08 ± 11.85	129.81 ± 13.99	95.67 ± 12.96
5-FU	22.26 ± 1.7	22.4 ± 4.1	15.4 ± 2.9	21.3 ± 4.2
Cisplatin	12.11 ± 2.5	18.22 ± 2.5	19.01 ± 1.5	25.13 ± 3.2

In summation, while the majority of synthesized compounds showed limited impact on the tested cell lines, compound 4j demonstrated impressive and consistent efficacy across all cell lines, making it a promising candidate for further investigation as an antiproliferative agent.

Moreover, 4j demonstrated a superior selectivity index (SI) across all the tested strains enlisted in Table 3. The SI was determined for each compound by employing the formula: SI = (IC_50_ for normal cell line FHC)/(IC_50_ for respective cancerous cell line). A favourable SI value > 1.0 signifies that the drug is more effective against tumor cells compared to its toxicity on normal cells. Comparing 4j to standard 5-FU and cisplatin, it exhibits good selectivity for HCT116, OVCAR10, and 1205Lu cancer cell lines, respectively, with SI values of 2.12, 2.40, and 1.68.

**Table 3:** The SI values of the synthesized compounds against HCT116, OVCAR10, 1205Lu, and FHC cell lines

Compound	IC_50_ (μM)				SI		
	HCT116	OVCAR10	1205Lu	FHC	HCT116	OVCAR10	1205Lu
4j	9.63 ± 1.3	8.56 ± 1.1	12.22 ± 1.7	20.56 ± 3.1	2.12	2.40	1.68
5-FU	22.26 ± 1.7	22.4 ± 4.1	15.4 ± 2.9	21.3 ± 4.2	0.95	0.95	1.38
Cisplatin	12.11 ± 2.5	18.22 ± 2.5	19.01 ± 1.5	25.13 ± 3.2	2.07	1.37	1.3

The inhibition data presented in Table 2 was used to derive the following structure–activity relationship (SAR):

(1) The presence of the benzyl group at the *N*-1 of the isatin core improved anti-proliferative activities as evidenced by the lack of activities of *N*-unsubstituted compounds 4m–o.

(2) The presence of Br at C-7 of the isatin core is preferred over H in the case of morpholine-substituted oxindole while negligible effects are observed in the case of piperidine-/pyrrolidine-substituted oxindoles.

(3) In the case of morpholine-substituted oxindoles, the antiproliferative activities follow the order: di-Br > Br > H = Cl. The order gets reversed in the case of piperidine/pyrrolidine-substituted oxindoles and the pattern followed is H > Br > Cl = di-Br.

**Fig. 5:**
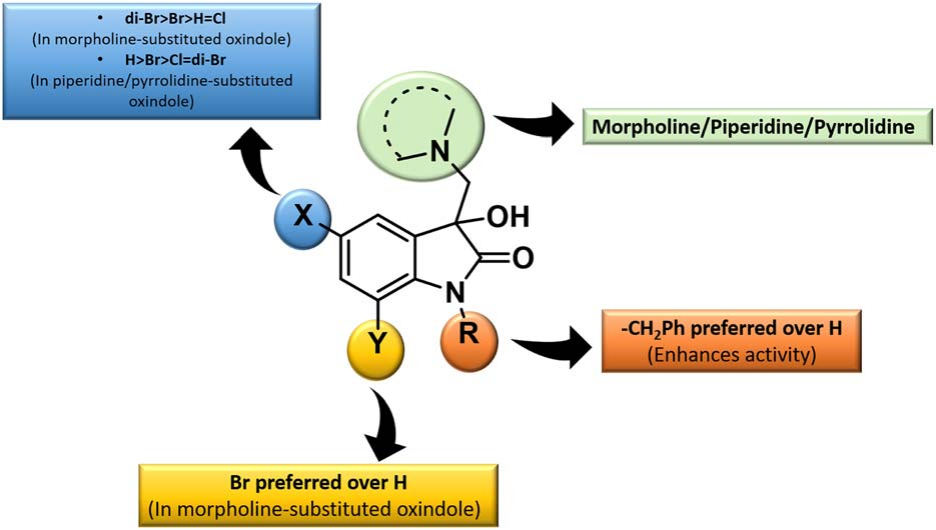
Generalised SAR for the synthesized 3-substituted-3-hydroxy-2-oxindoles.

The graphical representation of SAR is illustrated in Fig. 5.

#### Apoptosis assay

Apoptosis, a carefully regulated and anatomically distinct form of cell suicide, is a mechanism used by multicellular organisms to get rid of undesired, damaged, or diseased cells. Apoptosis is essential for sustaining adult tissues as well as for embryonic development, and its dysregulation can lead to autoimmune and neurological illnesses as well as cancer. Caspases, which may be activated by two distinct signalling routes are cysteine proteases that cleave after aspartate residues, cause apoptosis. There are two pathways at play: the intrinsic pathway, which is started by stress or damage inside the cell, and the extrinsic pathway, which is started extracellularly by the ligation of “death receptors,” a subset of the tumour necrosis factor (TNF) receptor complex. TNF-related apoptosis-inducing ligand (TRAIL) is a viable possibility for targeted cancer treatment because, in contrast to other TNF family ligands like CD95l, it may specifically cause apoptosis in tumour cells.^25,26^

**Fig. 6:**
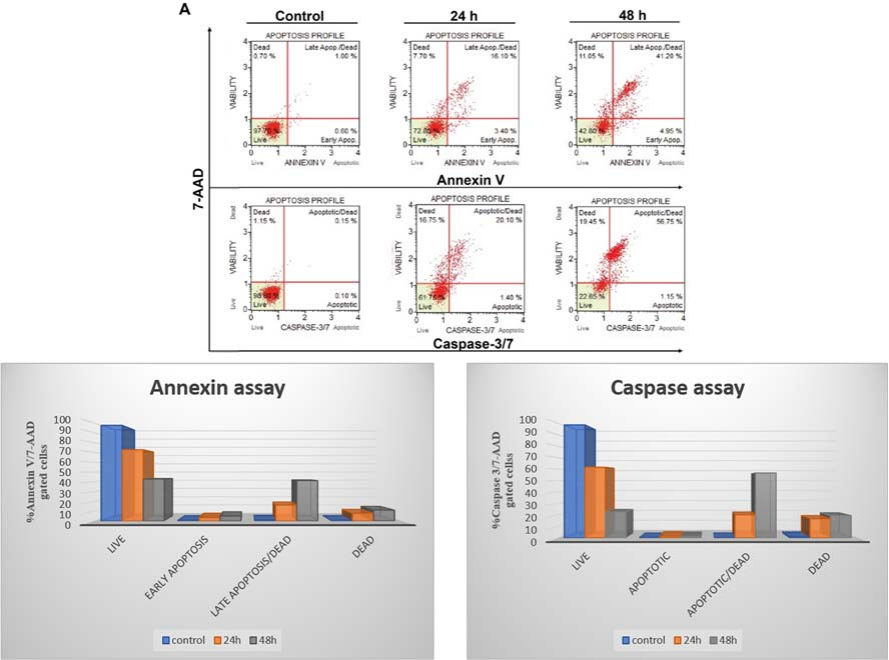
To assess the mode of cell death caused by compound 4j, apoptosis assays were performed. (A) The HCT-116 cells were treated with 10 μM of compound 4j for 24 and 48 h and subjected to Annexin V/7-AAD assay and Caspase 3/7 assays. The quantitative assessment of the apoptosis assays is presented as a bar graph in (B) and (C).

Based on cell viability data, 4j showed significant anti-proliferative activity against the tested cancer cells. We further carried out apoptosis experiments utilizing the caspase-3/7 7-AAD and Annexin V/7-AAD assays to evaluate the mode of cell death. As seen in Fig. 6, 10 μM of 4j was used for HCT-116 cells treatment for 24 and 48 h before the cells underwent apoptotic experiments. The data showed a significant increase in the apoptotic cell population after the treatment with 4j. In the Annexin V/7-AAD experiment, 4j treated HCT-116 cells showed 19.5% of apoptotic cells at 24 h and 46.15% at 48 h treatment. Similar to this, in HCT-116 cells treated with 4j for 24 and 48 h showed 21.5% and 57.9% of the cell population were found to be apoptotic by the Caspase 3/7 7-AAD assay, respectively. The results demonstrated that 4j has a stronger cytotoxic effect on HCT-116 cells which is mediated through the caspase apoptotic pathway.

### *In silico* studies

#### Molecular docking analysis

**Fig. 7:**
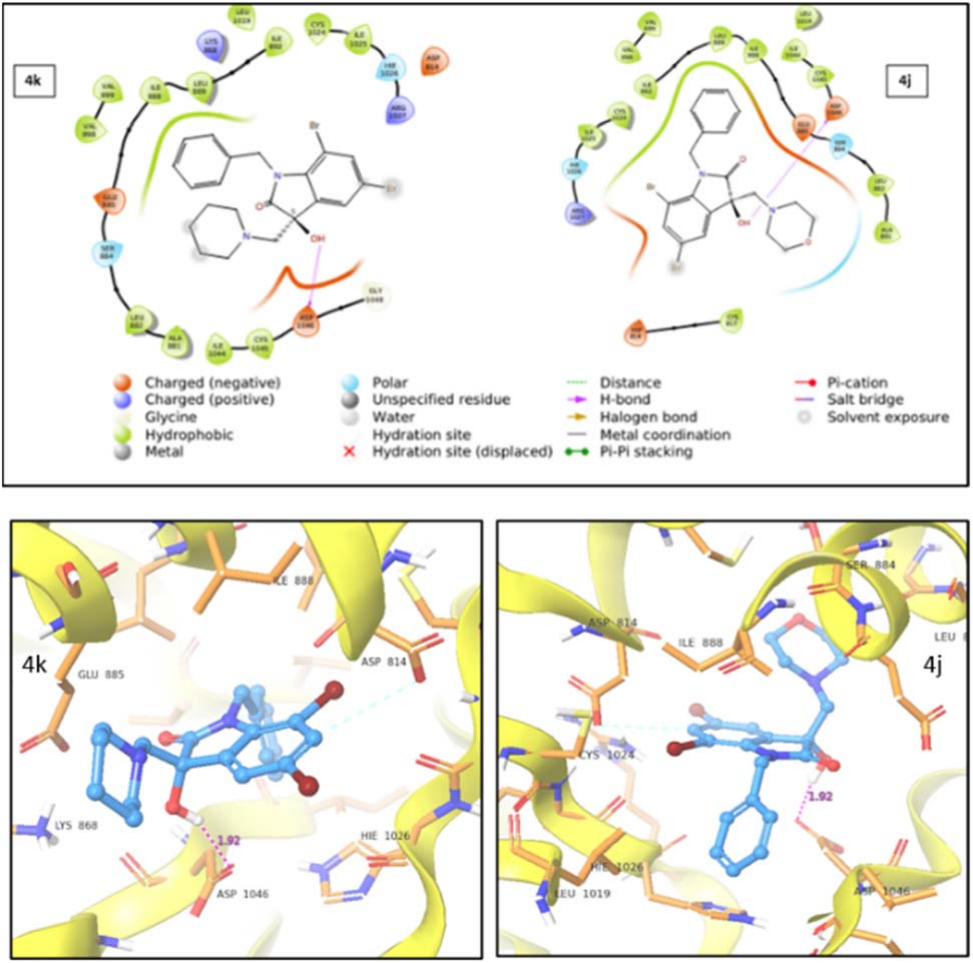
2D (top) and 3D (bottom) representation of VEGFR2 protein–ligand interaction for compound 4k and 4j.

We conducted *in silico* analyses employing three compounds- 4j (active), 4k (inactive), and 5-fluorouracil (reference)-against the VEGFR2 protein (PDB ID: 3VHE) to explain the observed anticancer activity and locate the unique binding core. The results of the docking tests showed that 4j had a good binding mode and was successfully docked inside the protein's active site as shown in Fig. 7. Further, the docking calculations of 4j, 4k and 5-fluorouracil are summarised in Table 4. The docking score for 4j was observed to be −6.228, whereas for 4k and the reference drug, it was found to be −3.857 and −4.198, respectively.

**Table 4:** Docking calculations of compounds 4j, 4k and 5-fluorouracil

Ligand	IC_50_			Docking score	XP GScore	Glide emodel
	HCT116	OVCAR10	1205Lu			
4j	9.63	8.56	12.22	−6.228	−6.252	−54.189
4k	109.53	91.23	89.85	−3.857	−5.830	−46.644
5-FU	22.26	22.4	15.4	−4.198	−4.916	−27.042

During studies two distinct types of interactions were identified. For both 4j and 4k compounds, an induced dipole–dipole interaction occurred between C6 and ASP814, alongside a hydrogen bonding interaction between –OH and ASP1046. Notably, in the case of 4j, an additional polar interaction was detected involving the oxygen within the morpholine group and the adjacent polar group, SER885, which is located in the active site. This specific interaction was absent in the case of 4k. The stronger protein–ligand interactions and a higher docking score for 4j compared to 4k provided support for the enhanced cytotoxic activity of 4j.

Thus, from the analyses of molecular docking data, it can be inferred that the active compound 4j outperforms the reference drug, 5-fluorouracil with notable protein interactions. Consequently, the suggested mechanism of action for 4j likely correlates with its cytotoxic effects against the VEGFR2 protein.

#### ADMET analysis

ADMET is an acronym that represents the vital processes of Absorption, Distribution, Metabolism, Excretion, and Toxicity. These processes play a pivotal role in determining a drug's pharmacokinetics and pharmacodynamics. Understanding these aspects is crucial for comprehending a drug's behavior within the body and assessing its effectiveness and safety. The ADMET descriptor module of small molecules was utilized for ADMET prediction, accessible at http://www.swissadme.ch/ and http://biosig.unimelb.edu.au/pkcsm/prediction. The acquired values are presented in Table S1 of the ESI.†

The ADMET studies for compounds 4a, 4d, 4j and 4m predicted that the number of rotatable bonds, hydrogen bond acceptors, and donors, and a total polar surface area lies within the recommended range with no violation of the Lipinski's rule of five. Among these, compounds 4a, 4d and 4m displayed log *P*_o/w_ values below 3, while compound 4j exhibited log *P*_o/w_ value above 3 suggesting 4j possessed noteworthy permeability through the cell membranes, indicating a well-balanced interplay between hydrophobic and hydrophilic traits highlighting its potential as a promising drug candidate. The compounds 4a and 4d were predicted to be P-glycoprotein II inhibitor while compound 4j was predicted to be both P-glycoprotein I and P-glycoprotein II inhibitor indicating good absorption and permeability. All the three compounds 4a, 4d and 4j can act as P-glycoprotein substrates while 4m cannot. Furthermore, all compounds showed high human intestinal absorption, indicating high oral bioavailability and affinity for interacting with target cells. The log BB (blood–brain barrier permeability) values suggested that these compounds exhibited substantial potential to cross the blood–brain barrier and enter the central nervous system (CNS). In conclusion, the ADMET studies predicted good oral absorption, permeability, and excellent intestinal bioavailability for the compounds.

## Experimental

### General information

^1^H NMR and ^13^C NMR spectra were recorded in deuterochloroform (CDCl_3_) and dimethylsulfoxide (DMSO-*d*_6_) with Jeol 400 (400 MHz) and Bruker 500 (500 MHz) spectrometer using TMS as an internal standard. Chemical shift values are expressed as parts per million downfield from TMS and *J* values are in hertz. Splitting patterns are indicated as s: singlet, d: doublet, t: triplet, m: multiplet, dd: double doublet, ddd: doublet of a doublet of a doublet, and br: broad peak. High resolution mass spectra were recorded on a Bruker-micrOTOF-Q II spectrometer.

#### General procedure for synthesis of C-5 substituted *N*-benzylated isatin 2(a–d)

To a stirred solution of substituted isatin 1(a–d) (1 mmol) in anhydrous DMF, sodium hydride (1.3 mmol) was added. The reaction mixture was allowed to stir for half an hour at 0 °C. The change of color from orange to dark purple confirmed the formation of anionic intermediate, subsequently benzyl bromide (1.2 mmol) was added to the reaction mixture. Reaction mixture was then allowed to stir for 1 h. Completion of the reaction was confirmed by TLC. After the completion of reaction, ice was added to the reaction mixture resulting in the precipitation of product from reaction mixture. The product was then filtered and dried to get 2(a–d) in good yields (not here). The crude product mixture was then purified by column chromatography on silica gel (60–120 mesh) using ethyl acetate : hexane (15 : 85) mixture as the eluent to afford the pure product.

#### General procedure for synthesis of spiro[indoline-3,2′-oxiran]-2-one (3e) and 1-benzylspiro[indoline-3,2′-oxiran]-2-one 3(a–d)

A mixture of trimethylsulfoxonium iodide (1.2 mmol) and sodium hydride (1.0 mmol) in dry DMF was stirred at 0 °C for 30 minutes. Isatin (1.0 mmol) in dry DMF (5 mL) as added dropwise to this solution over 10 minutes. The progress of the reaction was monitored by TLC. After completion of the reaction, ice was added to the reaction mixture resulting in the precipitation of the desired product from the reaction mixture. The product was then filtered and dried to get 3e in good yields. Similarly, *N*-benzylisatin (1.0 mmol) in dry DMF (5 mL) as added dropwise to the solution of Trimethylsulfoxonium iodide (1.2 mmol) and sodium hydride (1.0 mmol) in dry DMF over 10 minutes. The progress of the reaction was monitored by TLC. After completion of the reaction, ice was added to the reaction mixture resulting in the precipitation of the desired product from the reaction mixture. The product was then filtered and dried to get 3(a–d) in good yields.

#### General procedure for synthesis of 3-substituted-3-hydroxy-2-oxindoles derivatives 4(a–o)

To a well stirred solution of spiro-epoxy isatin 3(a–d) (1.0 mmol) in dry carbinol, secondary amines (piperidine/morpholine/pyrrolidine) (2.0 mmol) were added and the reaction mixture was stirred for 2 h. Upon completion of reaction, as monitored by TLC, solvent was removed under reduced pressure. The reaction mixture was extracted with ethyl acetate (3 × 10 mL). Combined organic layers were dried over anhydrous sodium sulphate and concentrated under reduced pressure. The reaction mixture was purified *via* column chromatography using ethyl acetate : hexane (40 : 60) to yield target compounds 4(a–o) in appreciable yields.

#### 1-Benzyl-3-hydroxy-3-(morpholinomethyl)indolin-2-one (4a)

Pale yellow; mp: 190–192 °C; ^1^H NMR (500 MHz, CDCl_3_) *δ* 7.38–7.34 (m, 5H, Ar-H), 7.30–7.28 (m, 1H, Ar-H), 7.23 (t, *J* = 7.7 Hz, 1H, Ar-H), 7.07 (t, *J* = 7.4 Hz, 1H, Ar-H), 6.71 (d, *J* = 7.8 Hz, 1H, Ar-H), 5.07 (d, *J* = 15.7 Hz, 1H, N–C**H**_2_–Ph), 4.72 (d, *J* = 15.8 Hz, 1H, N–C**H**_2_–Ph), 3.67–3.58 (m, 4H, **H**_2_C–O–C**H**_2_), 2.93 (d, *J* = 13.6 Hz, 1H, N–C**H**_2_–C(OH)), 2.88 (d, *J* = 13.7 Hz, 1H, N–C**H**_2_–C(OH)), 2.73–2.68 (m, 2H, **H**_2_C–N–H_2_C), 2.59–2.53 (m, 2H, H_2_C–N–**H**_2_C). ^13^C NMR (100 MHz, CDCl_3_) *δ* 177.46 (C=O), 142.23, 134.55, 128.85, 128.82, 127.78 (2C), 126.68, 126.25 (2C), 123.10, 121.98, 108.35, 73.66 (C–OH), 66.12 (2C), 63.52, 54.18 (2C), 42.77. HRMS calcd for C_20_H_22_N_2_O_3_ [M]^+^ 338.1630 and found 338.1642.

#### 1-Benzyl-3-hydroxy-3-(piperidin-1-ylmethyl)indolin-2-one (4b)

Pale yellow; mp: 139–141 °C; ^1^H NMR (400 MHz, CDCl_3_) *δ* 7.33–7.29 (m, 5H, Ar-H), 7.26–7.24 (m, 1H, Ar-H), 7.16 (td, *J* = 7.8, 1.2 Hz, 1H, Ar-H), 7.04–6.99 (m, 1H, Ar-H), 6.64 (d, 1H, Ar-H), 5.01 (d, *J* = 15.8 Hz, 1H, N–C**H**_2_–Ph), 4.70 (d, *J* = 15.8 Hz, 1H, N–C**H**_2_–Ph), 2.85 (d, *J* = 13.7 Hz, 1H, N–C**H**_2_–C(OH)), 2.78–2.70 (m, 3H, N–C**H**_2_–C(OH) + **H**_2_C–N–CH_2_), 2.58–2.50 (m, 2H, H_2_C–N–C**H**_2_), 1.60–1.51 (m, 4H, –C**H**_2_–CH_2_–C**H**_2_), 1.43–1.36 (m, 2H, CH_2_–C**H**_2_–CH_2_). ^13^C NMR (100 MHz, CDCl_3_) *δ* 178.87 (C=O), 143.12, 135.76, 130.79, 129.56, 128.82 (2C), 127.65, 127.30 (2C), 124.09, 122.98, 109.29, 74.12 (C–OH), 64.67, 56.53 (2C), 43.76, 26.36 (2C), 23.85. HRMS calcd for C_21_H_24_N_2_O_2_ [M]^+^ 336.1838 and found 336.1851.

#### 1-Benzyl-3-hydroxy-3-(pyrrolidin-1-ylmethyl)indolin-2-one (4c)

Pale yellow; mp: 169–171 °C; ^1^H NMR (500 MHz, CDCl_3_) *δ* 7.39 (d, *J* = 7.3 Hz, 1H, Ar-H), 7.33 (d, *J* = 5.7 Hz, 4H, Ar-H), 7.29–7.27 (m, 1H, Ar-H), 7.20 (t, *J* = 7.6 Hz, 1H, Ar-H), 7.06 (t, *J* = 7.4 Hz, 1H, Ar-H), 6.66 (d, *J* = 7.8 Hz, 1H, Ar-H), 5.14 (d, *J* = 15.8 Hz, 1H, N–C**H**_2_–Ph), 4.68 (d, *J* = 15.8 Hz, 1H, N–C**H**_2_–Ph), 3.17 (d, *J* = 13.1 Hz, 1H, N–C**H**_2_–C(OH)), 2.97 (d, *J* = 13.1 Hz, 1H, N–C**H**_2_–C(OH)), 2.75–2.69 (m, 2H, **H**_2_C–N–CH_2_), 2.68–2.62 (m, 2H, H_2_C–N–C**H**_2_), 1.79–1.71 (m, 4H, C**H**_2_–C**H**_2_). ^13^C NMR (100 MHz, CDCl_3_) *δ* 178.73 (C=O), 143.18, 135.75, 130.51, 129.63, 128.76 (2C), 127.59, 127.16 (2C), 124.18, 122.93, 109.31, 74.99 (C–OH), 62.77, 56.12 (2C), 43.73, 24.28 (2C). HRMS calcd for C_20_H_22_N_2_O_2_ [M]^+^ 322.1681 and found 322.1696.

#### 1-Benzyl-5-bromo-3-hydroxy-3-(morpholinomethyl)indolin-2-one (4d)

Pale yellow; mp: 198–200 °C; ^1^H NMR (500 MHz, CDCl_3_) *δ* 7.49 (s, 1H, Ar-H), 7.33 (dd, *J* = 13.9, 7.0 Hz, 6H, Ar-H), 6.58 (d, *J* = 8.3 Hz, 1H, Ar-H), 5.03 (d, *J* = 15.8 Hz, 1H, N–C**H**_2_–Ph), 4.72 (d, *J* = 15.7 Hz, 1H, N–C**H**_2_–Ph), 3.67–3.64 (m, 4H, **H**_2_C–O–C**H**_2_), 2.91 (d, *J* = 13.7 Hz, 1H, N–C**H**_2_–C(OH)), 2.84 (d, *J* = 13.7 Hz, 1H, N–C**H**_2_–C(OH)), 2.78–2.72 (m, 2H, H_2_C–N– **H**_2_C), 2.61–2.54 (m, 2H, **H**_2_C–N– H_2_C). ^13^C NMR (125 MHz, CDCl_3_) *δ* 176.85 (C=O), 141.19, 134.05, 131.60, 130.98, 127.89 (2C), 126.88, 126.49, 126.19 (2C), 114.70, 109.85, 73.58 (C–OH), 66.09 (2C), 63.38, 54.20 (2C), 42.86. HRMS calcd for C_20_H_21_BrN_2_O_3_ [M]^+^ 416.0736, [M + 2]^+^ 418.0715 and found 416.0748 and 418.0731.

#### 1-Benzyl-5-bromo-3-hydroxy-3-(piperidin-1-ylmethyl)indolin-2-one (4e)

Pale yellow; mp: 189–191 °C; ^1^H NMR (500 MHz, CDCl_3_) *δ* 7.46 (s, 1H, Ar-H), 7.35–7.28 (m, 6H, Ar-H), 6.55 (d, *J* = 8.0 Hz, 1H, Ar-H), 4.99 (d, *J* = 15.7 Hz, 1H, N–C**H**_2_–Ph), 4.74 (d, *J* = 15.7 Hz, 1H, N–C**H**_2_–Ph), 2.87 (d, *J* = 13.7 Hz, 1H, N–C**H**_2_–C(OH)), 2.82–2.72 (m, 3H, N–C**H**_2_–C(OH) + **H**_2_C–N–CH_2_), 2.59 (s, 2H, H_2_C–N–C**H**_2_), 1.66–1.57 (m, 4H, **H**_2_C–CH_2_–C**H**_2_), 1.49–1.41 (m, 2H, H_2_C–C**H**_2_–CH_2_). ^13^C NMR (100 MHz, CDCl_3_) *δ* 178.34 (C=O), 142.09, 135.24, 132.93, 132.33, 128.92 (2C), 127.84, 127.43, 127.24 (2C), 115.70, 110.79, 73.93 (C–OH), 64.49, 56.54 (2C), 43.85, 26.35 (2C), 23.78. HRMS calcd for C_21_H_23_BrN_2_O_2_ [M]^+^ 414.0943, [M + 2]^+^ 416.0922 and found 414.0956 and 416.0935.

#### 1-Benzyl-5-bromo-3-hydroxy-3-(pyrrolidin-1-ylmethyl)indolin-2-one (4f)

Pale yellow; mp: 144–146 °C; ^1^H NMR (500 MHz, CDCl_3_) *δ* 7.40 (d, *J* = 1.9 Hz, 1H, Ar-H), 7.24–7.22 (m, 1H, Ar-H), 7.22–7.20 (m, 3H, Ar-H), 7.19 (s, 2H, Ar-H), 6.43 (d, *J* = 8.3 Hz, 1H, Ar-H), 5.00 (d, *J* = 15.9 Hz, 1H, N–C**H**_2_–Ph), 4.58 (d, *J* = 15.9 Hz, 1H, N–C**H**_2_–Ph), 3.05 (d, *J* = 13.2 Hz, 1H, N–C**H**_2_–C(OH)), 2.87 (d, *J* = 13.2 Hz, 1H, N–C**H**_2_–C(OH)), 2.67–2.62 (m, 2H, **H**_2_C–N–CH_2_), 2.58–2.54 (m, 2H, H_2_C–N–C**H**_2_), 1.67 (t, *J* = 6.4 Hz, 4H, C**H**_2_–C**H**_2_). ^13^C NMR (125 MHz, CDCl_3_) *δ* 177.10 (C=O), 141.09, 134.16, 131.58, 131.32, 127.78 (2C), 126.71, 126.43, 126.03 (2C), 114.59, 109.73, 73.79 (C–OH), 61.62, 55.10 (2C), 42.75, 23.25 (2C). HRMS calcd for C_20_H_21_BrN_2_O_2_ [M]^+^ 400.0786, [M + 2]^+^ 402.0766 and found 400.0798 and 402.0779.

#### 1-Benzyl-5-chloro-3-hydroxy-3-(morpholinomethyl)indolin-2-one (4g)

Pale yellow; mp: 191–193 °C; ^1^H NMR (500 MHz, CDCl_3_) *δ* 7.28–7.21 (m, 6H, Ar-H), 7.10 (d, *J* = 7.6 Hz, 1H, Ar-H), 6.53 (d, *J* = 7.9 Hz, 1H, Ar-H), 4.94 (d, *J* = 15.6 Hz, 1H, N–C**H**_2_–Ph), 4.63 (d, *J* = 15.7 Hz, 1H, N–C**H**_2_–Ph), 3.64–3.49 (m, 4H, **H**_2_C–O–C**H**_2_), 2.82 (d, *J* = 13.5 Hz, 1H, N–C**H**_2_–C(OH)), 2.75 (d, *J* = 13.7 Hz, 1H, N–C**H**_2_–C(OH)), 2.69–2.62 (m, 2H, H_2_C–N–C**H**_2_), 2.52–2.44 (m, 2H, **H**_2_C–N–CH_2_). ^13^C NMR (125 MHz, CDCl_3_) *δ* 177.96 (C=O), 141.66, 135.06, 131.58, 129.69, 128.89 (2C), 128.49, 127.87, 127.19 (2C), 124.70, 110.37, 74.58 (C–OH), 67.10 (2C), 64.37, 55.19 (2C), 43.88. HRMS calcd for C_20_H_21_ClN_2_O_3_ [M]^+^ 372.1241, [M + 2]^+^ 374.1211 and found 372.1257 and 374.1216.

#### 1-Benzyl-5-chloro-3-hydroxy-3-(pyrrolidin-1-ylmethyl)indolin-2-one (4i)

Pale yellow; mp: 121–124 °C; ^1^H NMR (500 MHz, CDCl_3_) *δ* 7.27 (d, *J* = 1.6 Hz, 1H, Ar-H), 7.24–7.21 (m, 3H, Ar-H), 7.19 (s, 2H, Ar-H), 7.07 (d, *J* = 8.3 Hz, 1H, Ar-H), 6.48 (d, *J* = 8.3 Hz, 1H, Ar-H), 5.00 (d, *J* = 15.8 Hz, 1H, N–C**H**_2_–Ph), 4.59 (d, *J* = 15.9 Hz, 1H, N–C**H**_2_–Ph), 3.05 (d, *J* = 13.2 Hz, 1H, N–C**H**_2_–C(OH)), 2.87 (d, *J* = 13.2 Hz, 1H, N–C**H**_2_–C(OH)), 2.70–2.64 (m, 2H, **H**_2_C–N– CH_2_), 2.61–2.54 (m, 2H, H_2_C–N–**CH**_2_), 1.70–1.66 (m, 4H, **H**_2_C–C**H**_2_). ^13^C NMR (125 MHz, CDCl_3_) *δ* 178.16 (C=O), 141.58, 135.20, 132.20, 129.42, 128.78 (2C), 128.35, 127.71, 127.04 (2C), 124.69, 110.23, 74.79 (C–OH), 62.59, 56.11 (2C), 43.78, 24.25 (2C). HRMS calcd for C_20_H_21_ClN_2_O_2_ [M]^+^ 356.1292, [M + 2]^+^ 358.1262 and found 356.1308 and 358.1278.

#### 1-Benzyl-5,7-dibromo-3-hydroxy-3-(morpholinomethyl)indolin-2-one (4j)

Reddish brown; mp: 146–148 °C; ^1^H NMR (400 MHz, CDCl_3_) *δ* 7.53 (d, *J* = 1.8 Hz, 1H, Ar-H), 7.42 (d, *J* = 1.8 Hz, 1H, Ar-H), 7.31 (d, *J* = 6.9 Hz, 1H, Ar-H), 7.28 (s, 1H, Ar-H), 7.25 (t, *J* = 3.3 Hz, 1H, Ar-H), 7.21 (d, *J* = 7.6 Hz, 2H, Ar-H), 5.31 (s, 2H, N–C**H**_2_–Ph), 3.63 (t, *J* = 4.5 Hz, 4H, **H**_2_C–O–C**H**_2_), 2.87 (d, *J* = 13.8 Hz, 1H, N–C**H**_2_–C(OH)), 2.78 (d, *J* = 13.8 Hz, 1H, N–C**H**_2_–C(OH)), 2.74–2.68 (m, 2H, **H**_2_C–N–CH_2_), 2.55–2.49 (m, 2H, H_2_C–N–C**H**_2_). ^13^C NMR (100 MHz, CDCl_3_) *δ* 177.69 (C=O), 139.00, 136.51, 135.74, 133.80, 127.65 (2C), 126.32, 125.70, 125.28 (2C), 115.04, 102.19, 72.86 (C–OH), 66.04 (2C), 63.60, 54.20 (2C), 43.51. HRMS calcd for C_20_H_20_Br_2_N_2_O_3_ [M]^+^ 493.9841, [M + 2]^+^ 495.9820, [M + 4]^+^ 497.9800 and found 493.9864, 495.9841 and 497.9818.

#### 1-Benzyl-5,7-dibromo-3-hydroxy-3-(piperidin-1ylmethyl)indolin-2-one (4k)

Reddish brown; mp: 170–172 °C; ^1^H NMR (400 MHz, CDCl_3_) *δ* 7.28 (d, *J* = 5.8 Hz, 3H, Ar-H), 7.24 (s, 1H, Ar-H), 7.21 (s, 1H, Ar-H), 7.14 (d, *J* = 7.2 Hz, 2H, Ar-H), 5.57–5.49 (m, 2H, N–C**H**_2_–Ph), 4.50–4.43 (m, 1H, N–C**H**_2_–C(OH)), 3.72–3.66 (m, 1H, N–C**H**_2_–C(OH)), 3.65–2.57 (m, 2H, **H**_2_C–N–CH_2_), 1.85–1.78 (m, 4H, **H**_2_C–CH_2_–C**H**_2_), 1.78–1.71 (m, 2H, H_2_C–N–C**H**_2_), 1.66–1.57 (m, 2H, H_2_C–C**H**_2_–CH_2_). ^13^C NMR (100 MHz, CDCl_3_) *δ* 165.11 (C=O), 146.39, 138.98, 132.58, 129.17, 128.45 (2C), 126.67, 126.21 (2C), 115.66, 113.37, 102.04, 91.23 (C–OH), 57.84, 52.30, 44.01 (2C), 26.39, 23.75 (2C). HRMS calcd for C_21_H_22_Br_2_N_2_O_2_ [M]^+^ 492.0048, [M + 2]^+^ 494.0028, [M + 4]^+^ 496.0007 and found 492.0062, 494.0031 and 496.0019.

#### 3-Hydroxy-3-(morpholinomethyl)indolin-2-one (4m)

Pale yellow; mp: 126–128 °C; ^1^H NMR (500 MHz, CDCl_3_) *δ* 8.12 (s, 1H, NH-exchangeable with D_2_O), 7.35 (d, *J* = 7.3 Hz, 1H, Ar-H), 7.30–7.24 (m, 1H, Ar-H), 7.08 (t, *J* = 7.5 Hz, 1H, Ar-H), 6.87 (d, *J* = 7.7 Hz, 1H, Ar-H), 3.73–3.63 (m, 4H, **H**_2_C–O–C**H**_2_), 2.88 (d, *J* = 13.8 Hz, 1H, N–C**H**_2_–C(OH)), 2.81–2.74 (m, 3H, N–C**H**_2_–C(OH) + **H**_2_C–N–CH_2_), 2.60–2.53 (m, 2H, H_2_C–N–C**H**_2_). ^13^C NMR (125 MHz, CDCl_3_) *δ* 180.54 (C=O), 140.88, 130.36, 129.83, 124.47, 123.01, 110.08, 74.78 (C–OH), 67.22 (2C), 64.21, 55.12 (2C). HRMS calcd for C_13_H_16_N_2_O_3_ [M]^+^ 248.1161 and found 248.1176.

#### 3-Hydroxy-3-(piperidin-1-ylmethyl)indolin-2-one (4n)

Pale yellow; mp: 154–156 °C; ^1^H NMR (500 MHz, CDCl_3_) *δ* 7.79 (s, 1H, NH-exchangeable with D_2_O), 7.23 (d, *J* = 7.4 Hz, 1H, Ar-H), 7.16 (t, *J* = 7.7 Hz, 1H, Ar-H), 6.97 (t, *J* = 7.5 Hz, 1H, Ar-H), 6.76 (d, *J* = 7.7 Hz, 1H, Ar-H), 2.77–2.68 (m, 3H, N–C**H**_2_–C(OH) + **H**_2_C–N–CH_2_), 2.62 (d, *J* = 13.8 Hz, 1H, N–C**H**_2_–C(OH)), 2.53–2.46 (m, 2H, H_2_C–N–C**H**_2_), 1.56–1.49 (m, 4H, **H**_2_C–CH_2_–C**H**_2_), 1.38–1.32 (m, 2H, H_2_C–C**H**_2_–CH_2_). ^13^C NMR (125 MHz, CDCl_3_) *δ* 180.84 (C=O), 140.73, 131.27, 129.50, 124.36, 122.91, 109.94, 73.97 (C–OH), 64.33, 56.40 (2C), 26.30 (2C), 23.73. HRMS calcd for C_14_H_18_N_2_O_2_ [M]^+^ 246.1368 and found 246.1383.

#### 3-Hydroxy-3-(pyrrolidin-1-ylmethyl)indolin-2-one (4o)

Pale yellow; mp: 170–172 °C; ^1^H NMR (500 MHz, CDCl_3_) *δ* 8.13 (s, 1H, NH-exchangeable with D_2_O), 7.35 (d, *J* = 7.4 Hz, 1H, Ar-H), 7.26 (t, *J* = 7.7 Hz, 1H, Ar-H), 7.07 (t, *J* = 7.5 Hz, 1H, Ar-H), 6.86 (d, *J* = 7.7 Hz, 1H, Ar-H), 3.05 (d, *J* = 13.4 Hz, 1H, N–C**H**_2_–C(OH)), 2.92 (d, *J* = 13.4 Hz, 1H, N–C**H**_2_–C(OH)), 2.86–2.80 (m, 2H, **H**_2_C–N–CH_2_), 2.71–2.65 (m, 2H, H_2_C–N–C**H**_2_), 1.80–1.75 (m, 4H, **H**_2_C–C**H**_2_). ^13^C NMR (125 MHz, CDCl_3_) *δ* 180.80 (C=O), 140.89, 130.90, 129.58, 124.48, 122.85, 110.04, 74.70 (C–OH), 62.49, 56.15 (2C), 24.24 (2C). HRMS calcd for C_13_H_16_N_2_O_2_ [M]^+^ 232.1212 and found 232.1231.

## Materials and methods

### Cell culture

HCT116, OVCAR 10, 1205Lu, and FHC cell lines were procured from American Type Culture Collection (ATCC, Manassas, VA, USA). All the cells were grown based on the recommendations by ATCC at 37 °C in a humidified atmosphere with CO_2_. Stock solutions of compounds were made up and delivered to cells in DMSO.

### Cell viability assay

The cell viability assay was performed as described earlier^27^ HCT116, OVCAR 10, 1205Lu, and FHC adherent cell lines were seeded in 96-well plate at a density of 5 × 10^3^ cells per well. Next day, the cells were treated with the respective compounds at different doses for 48 h in triplicates. At the end of the treatment duration, the media were removed and fresh media were added containing 20 μL of [3-(4,5-dimethylthiazol-2-yl)-5-(3-carboxymethoxyphenyl)-2-(4-sulfophenyl)-2*H*-tetrazolium] (MTS) reagent per well (Promega, WI, USA). The optical densities of the solubilized formazan crystals were read with a 96-well multi-scanner (Dynex Technologies, MRX Revelation; Chantilly, VA, USA) at 490 nm and 630 nm, and the normalized percent viability was calculated. The IC_50_ values were calculated using non-linear regression (curve fit).

### Apoptosis assays

The apoptosis assay using Muse Annexin V/7-AAD and Caspase 3/7 7-AAD assay kit (EMD Millipore, Darmstadt, Germany) was performed following manufacturer's protocol as described earlier.^28^ Briefly, the cells were seeded in 6-well plate at a density of 1 × 10^5^ cells per well. After 24 h incubation, the cells were treated with 10 μM of compound 4j. The cells were incubated for 24 and 48 h. At the end of the treatment period, the cells were harvested using enzyme free cell dissociation buffer. The samples were treated with the respective dyes based on the manufacturer's protocol. The samples were further analysed in Muse Cell Analyzer (Merck Millipore, Darmstadt, Germany).

### Single crystal XRD

#### Single crystals of C_20_H_22_N_2_O_3_

A suitable crystal were selected for indexing and the intensity data were recorded at 296 K on a Bruker DUO diffractometer equipped with a Mo Incoatec Microfocus Source IμS (*λ* = 0.71073 Å). Using Olex2,^29^ the structure was solved with the XS structure solution program using Direct Methods and refined with the XL^30^ refinement package using Least Squares minimisation. All non-hydrogen atoms were refined anisotropically and all H atoms linked to N and O atoms were located from the electron density difference map and refined riding on their parent atoms. CCDC-2289774 (for 4a) contain the supplementary crystallographic data for this paper.†

### Molecular docking protocols

The molecular docking was performed using Schrodinger (Schrödinger Release 2022-1: Maestro, Schrödinger, LLC, New York, NY). Crystal co-ordinates of VEGFR2 protein (PDB ID: 3VHE) were downloaded from the protein data bank (https://www.rcsb.org). In the first step, bond orders were assigned and hydrogens were added by pre-process option. All water molecules were deleted. The heteroatoms are ionized by epic at biological pH to consider the protein permeability and drug solubility and then the H-bonds were optimized to reduce the steric clashes by histidine, aspartate, glutamate, and hydroxyl containing amino acids. Then complete protein structure was minimized by using OPLS 2005 force field. Ligand preparation is generally required because molecules lack 3D coordinates, ionization, stereochemistry and tautomers. Thus, before docking, the least energy state of ligand was needed to be prepared. Ligprep tool of Schrödinger was used to prepare the least energy state of ligand which converts 1D/2D structures to 3D. Finally, this ligand molecule was minimized with the help of the OPLS 2005 force field. For docking, the grids were generated by using the grid-based energy descriptor which had a default set of options with van der Waals radius of 1.0. The glide docking of the molecules was carried out using the previously prepared receptor grid and the ligand molecules. The favourable interactions between ligand molecules and the receptor were scored using Glide ligand docking program. All the docking calculations were performed using extra precision (XP) mode and OPLS-2005 force field. Thus H-bonding, hydrophobic interactions and π–π stacking between enzyme and compound were determined.^31–35^

## Conclusion

The present manuscript involves the synthesis of 3-substituted-3-hydroxy-2-oxindoles derivatives and their antiproliferative evaluation against HCT116, OVCAR 10, 1205Lu, and FHC cell lines. Among the synthesized compounds, morpholine-substituted di-bromo derivative 4j showed promising activity against all the tested cancer cell lines. With the IC_50_ values of 9.63 and 8.56 μM against HCT116 and OVCAR, respectively, it outperforms the 5-FU and cisplatin. The activity in the case of 1205Lu (IC_50_ = 12.22 μM) was also better than the reference drugs used. Furthermore, 4j demonstrated an increased induction of apoptotic cell death in the HCT116 cell line, thus confirming the involvement of caspase-mediated apoptotic mechanisms. Molecular docking studies shed light on the mode of action, revealing that the active compound surpasses the reference drug, 5-fluorouracil through notable protein interactions. These interactions are implicated in its cytotoxic effects, wherein binding with the VEGFR2 protein is particularly significant.

## Author contributions

Preeti – synthesized and analysed the data; AR – conducted the biological studies; Preeti and AA – conducted docking studies; NH and PR – conducted X-ray diffraction studies and finalized the draft; AS – analysed the biological data and finalized the draft; VK – conceptualized the work, compiled the data and finalized the draft.

## Conflicts of interest

There are no conflicts to declare.

## Supplementary Material

RA-013-D3RA05869G-s001

RA-013-D3RA05869G-s002

## References

[cit1] https://www.cancer.org/research/cancer-facts-statistics/all-cancer-facts-figures/2023-cancer-facts-figures.html, accessed on August 17, 2023

[cit2] Al-Sanea M. M., Chilingaryan G., Abelyan N., Sargsyan A., Hovhannisyan S., Gasparyan H., Gevorgyan S., Albogami S., Ghoneim M. M., Farag A. K., Mohamed A. A. (2021). Life.

[cit3] Madu C. O., Wang S., Madu C. O., Lu Y. (2020). J. Cancer.

[cit4] Wang X., Bove A. M., Simone G., Ma B. (2020). Front. Cell Dev. Biol..

[cit5] Modi S. J., Kulkarni V. M. (2019). Med. Drug Discovery.

[cit6] Lugano R., Ramachandran M., Dimberg A. (2020). Cell. Mol. Life Sci..

[cit7] Peng F. W., Liu D. K., Zhang Q. W., Xu Y. G., Shi L. (2017). Expert Opin. Ther. Pat..

[cit8] Farghaly T. A., Al-Hasani W. A., Abdulwahab H. G. (2021). Expert Opin. Ther. Pat..

[cit9] World Health Organization , Working to Overcome the Global Impact of Neglected Tropical Diseases: First WHO Report on Neglected Tropical Diseases, World Health Organization, Geneva, Switzerland, 2010, vol. 1

[cit10] Esmaeelian B., Abbott C. A., Le Leu R., Benkendorff K. (2013). Mar. Drugs.

[cit11] Kakkar R. (2019). MedChemComm.

[cit12] Chowdhary S., Arora A., Kumar V. (2022). Pharmaceuticals.

[cit13] Huang C. Y., Doyle A. G. (2014). Chem. Rev..

[cit14] Xuan J., He X. K., Xiao W. J. (2020). Chem. Soc. Rev..

[cit15] Sweeney J. B. (2002). Chem. Soc. Rev..

[cit16] Shankaraiah N., Sakla A. P., Laxmikeshav K., Tokala R. (2020). Chem. Rec..

[cit17] Zhu G., Bao G., Li Y., Sun W., Li J., Hong L., Wang R. (2017). Angew. Chem., Int. Ed..

[cit18] Peddibhotla S. (2009). Curr. Bioact. Compd..

[cit19] Sharma B., Saha S. T., Perumal S., Gu L., Ebenezer O., Singh P., Kaur M., Kumar V. (2020). ACS Omega.

[cit20] Chowdhary S., Raza A., Seboletswe P., Cele N., Sharma A. K., Singh P., Kumar V. (2023). J. Mol. Struct..

[cit21] Kumar S., Saini A., Kumar A., Raj R., Kumar V. (2022). J. Heterocycl. Chem..

[cit22] Shalini, Lata S., Saha S. T., Kaur M., Awolade P., Ebenezer O., Singh P., Kumar V. (2022). J. Mol. Struct..

[cit23] Hajra S., Maity S., Maity R. (2015). Org. Lett..

[cit24] Tak R. K., Gupta N., Kumar M., Kureshy R. I., Khan N. U. H., Suresh E. (2018). Eur. J. Org Chem..

[cit25] Dickens L. S., Boyd R. S., Jukes-Jones R., Hughes M. A., Robinson G. L., Fairall L., Schwabe J. W., Cain K., MacFarlane M. (2012). Mol. Cell.

[cit26] Li P., Nijhawan D., Budihardjo I., Srinivasula S. M., Ahmad M., Alnemri E. S., Wang X. (1997). Cell.

[cit27] Raza A., Singh A., Amin S., Spallholz J. E., Sharma A. K. (2022). Chem.-Biol. Interact..

[cit28] Ramos-Inza S., Encío I., Raza A., Sharma A. K., Sanmartín C., Plano D. (2022). Eur. J. Med. Chem..

[cit29] Dolomanov O. V., Bourhis L. J., Gildea R. J., Howard J. A., Puschmann H. (2009). J. Appl. Crystallogr..

[cit30] Sheldrick G. M. (2008). Acta Crystallogr., Sect. A: Found. Crystallogr..

[cit31] Greenwood J. R., Calkins D., Sullivan A. P., Shelley J. C. (2010). J. Comput.-Aided Mol. Des..

[cit32] Shelley J. C., Cholleti A., Frye L. L., Greenwood J. R., Timlin M. R., Uchimaya M. J. (2007). J. Comput.-Aided Mol. Des..

[cit33] Shivakumar D., Williams J., Wu Y., Damm W., Shelley J., Sherman W. (2010). J. Chem. Theory Comput..

[cit34] Jorgensen W. L., Maxwell D. S., Tirado-Rives J. (1996). J. Am. Chem. Soc..

[cit35] Jorgensen W. L., Tirado-Rives J. (1988). J. Am. Chem. Soc..

