# Supplementary material for: Stereo/regio-selective access to substituted 3-hydroxy-oxindoles with anti-proliferative assessment and in silico validation
Source: RSC Adv. 2023 Sep 27;13(41):28434–43. doi: 10.1039/d3ra05869g (PMC10523091; doi:10.1039/d3ra05869g)

Electronic Supplementary Material (ESI) for RSC Advances.
This journal is © The Royal Society of Chemistry 2023

Stereo/regio-selective access to substituted 3-hydroxy-oxindoles with anti-proliferative assessment and *in-silico* validation†

Preeti,[a] Asif Raza,[b] Amit Anand,[c] Natacha Henry,[d] Arun K. Sharma,[b] Pascal Roussel,[d] Vipan Kumar[a*]

[a]*Department of Chemistry, Guru Nanak Dev University, Amritsar, India*
[b]*Department of Pharmacology, Penn State Cancer Institute, The Pennsylvania State University College of Medicine, Hershey, PA 17033*
[c]*Department of Chemistry, Khalsa College, Amritsar, India*
[d]*Univ. Lille, CNRS, Centrale Lille, Univ. Artois, UMR 8181, Unité de Catalyse et Chimie du Solide (UCCS), F-59000 Lille, France*

Contents

1. Physiochemical and ADMET properties of compounds 4a, 4d, 4j and 4m
2. Scanned copies of ^{1}H and ^{13}C-NMR spectra of few representative compounds

Table S1: Physicochemical and ADMET properties of compounds **4a, 4d, 4j** and **4m**.

	**4a**	**4d**	**4j**	**4m**
**Absorption**				
Water solubility (log mol/L)	-2.855	-3.124	-3.411	-2.252
Caco2 permeability (log Papp in 10-6 cm/s)	1.189	0.886	0.885	1.117
Intestinal absorption (human) (% Absorbed)	93.868	92.941	91.328	86.619
Skin permeability (log Kp)	-2.745	-2.746	-2.746	-2.745
P-glycoprotein substrate	Yes	Yes	Yes	No
P-glycoprotein I inhibitor	No	No	Yes	No
P-glycoprotein II inhibitor	Yes	Yes	Yes	No
**Metabolism**				
CYP2D6 inhibitor	Yes	Yes	Yes	Yes
CYP3A4 inhibitor	Yes	Yes	Yes	No
CYP1A2 inhibitor	Yes	No	No	No
CYP2C19 Inhibitor	No	No	No	No
CYP2C9 inhibitor	No	No	No	No
CYP2D6 inhibitor	Yes	Yes	No	No
CYP3A4 inhibitor	No	No	No	No
**Excretion**				
Total Clearance (log ml/min/kg)	1.059	0.972	0.591	1.093
Renal OCT2 substrate	No	No	No	No
**Distribution**				
VDss (human) (log L/kg)	1.066	1.098	1.065	0.08
BBB permeability (log BB)	0.042	0.011	-0.04	-0.244
Fraction unbound (human) (Fu)	0.206	0.23	0.261	0.728
CNS permeability (log PS)	-2.55	-2.414	-2.266	-2.965
**Toxicity**				

Oral Rat Acute Toxicity (LD50) (mol/kg)	2.772	2.866	2.942	2.851
Hepatotoxicity	Yes	No	Yes	No
Skin Sensitisation	No	No	No	No
T.Pyriformis toxicity (log ug/L)	0.305	0.31	0.312	0.258
Minnow toxicity (log mM)	1.762	1.123	0.616	2.401
**Molecular weight**	338.40	417.30	496.19	248.28
**Log$P_{o/w}$**	1.7532	2.5157	3.2782	0.1585
**TPSA (Å)**	53.01	53.01	53.01	61.80
**No. of rotatable bonds**	4	4	4	2
**No. of HBA**	4	4	4	4
**No. of HBD**	1	1	1	1
**Molar refractivity**	101.98	109.68	117.38	72.60

2. Scanned copies of ^{1}H and ^{13}C NMR spectra:

1. **^{1}H NMR** 1-Benzyl-3-hydroxy-3-(morpholinomethyl)indolin-2-one (**4a**).

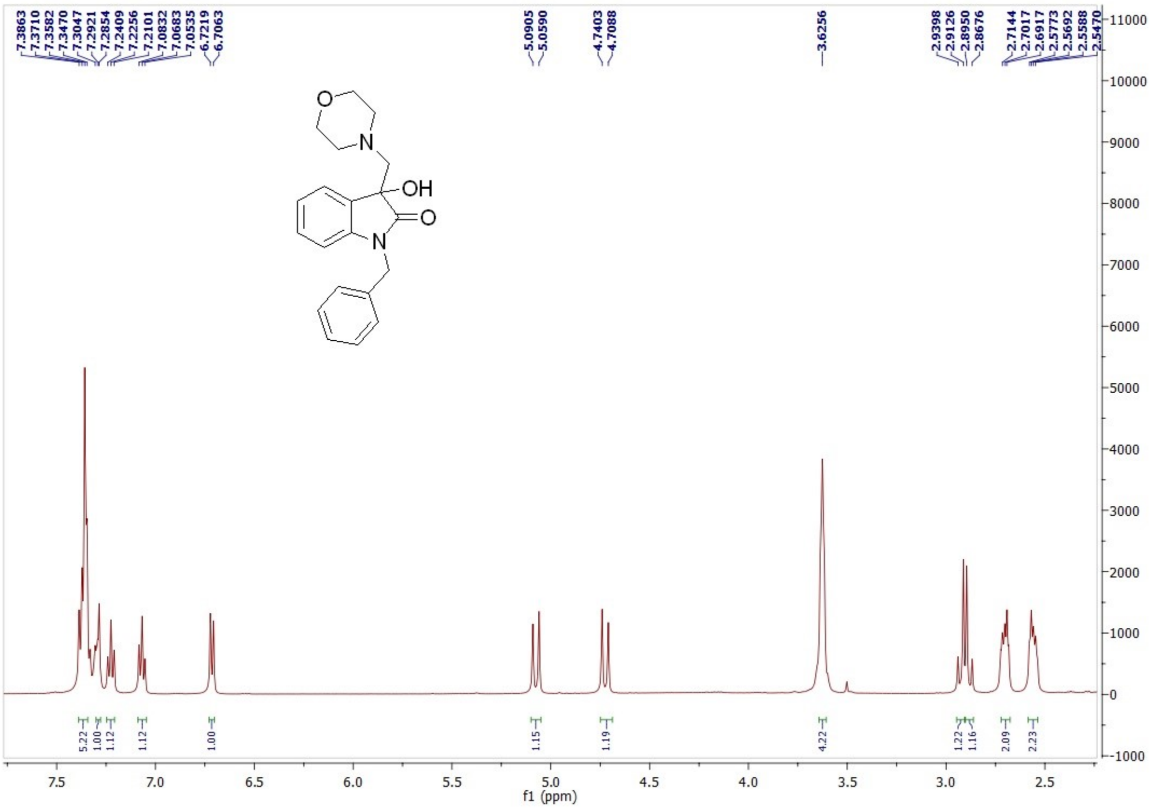

2. **^{13}C NMR** 1-Benzyl-3-hydroxy-3-(morpholinomethyl)indolin-2-one (**4a**)

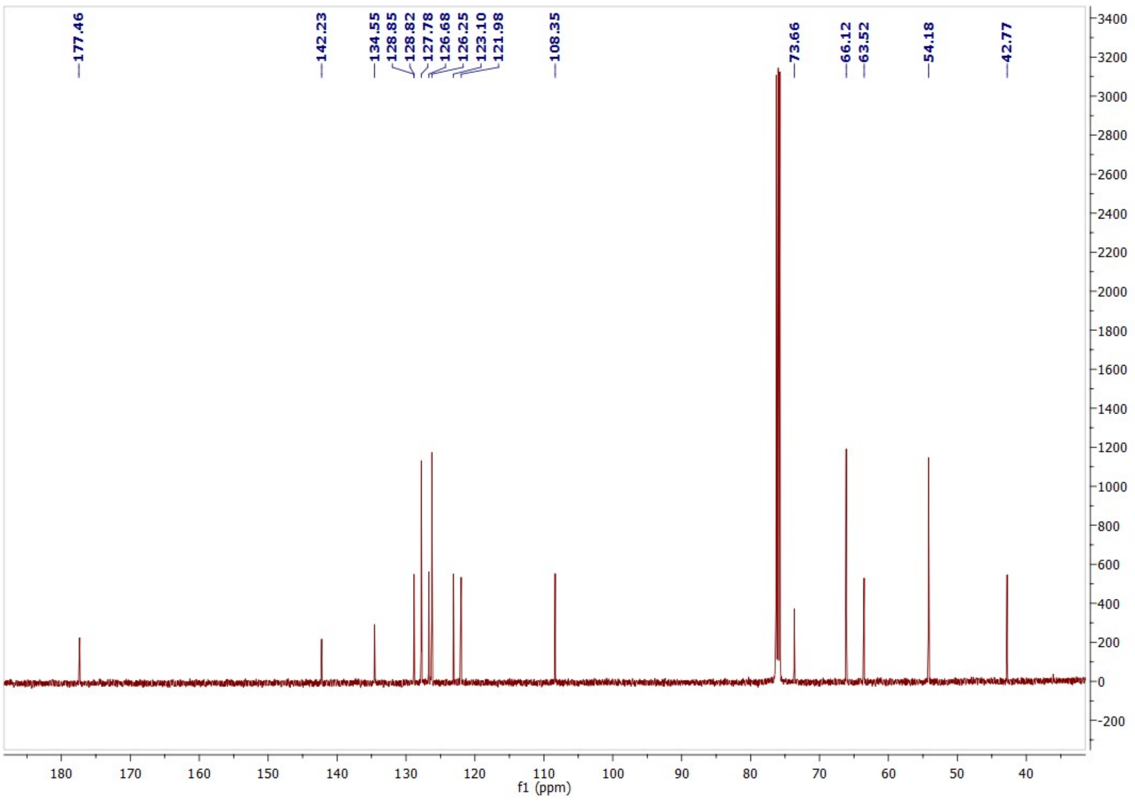

3. **^{1}H NMR** 1-Benzyl-3-hydroxy-3-(piperidin-1-ylmethyl)indolin-2-one (**4b**)

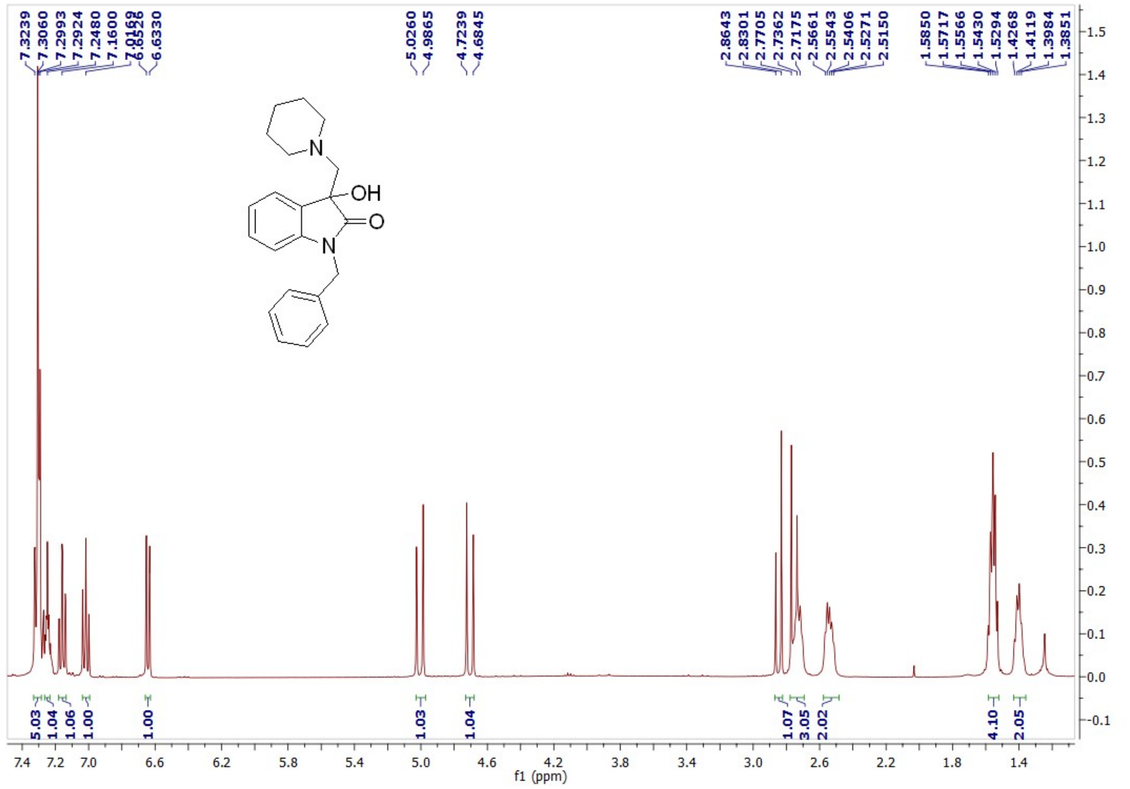

4. **^{13}C NMR** 1-Benzyl-3-hydroxy-3-(piperidin-1-ylmethyl)indolin-2-one (**4b**)

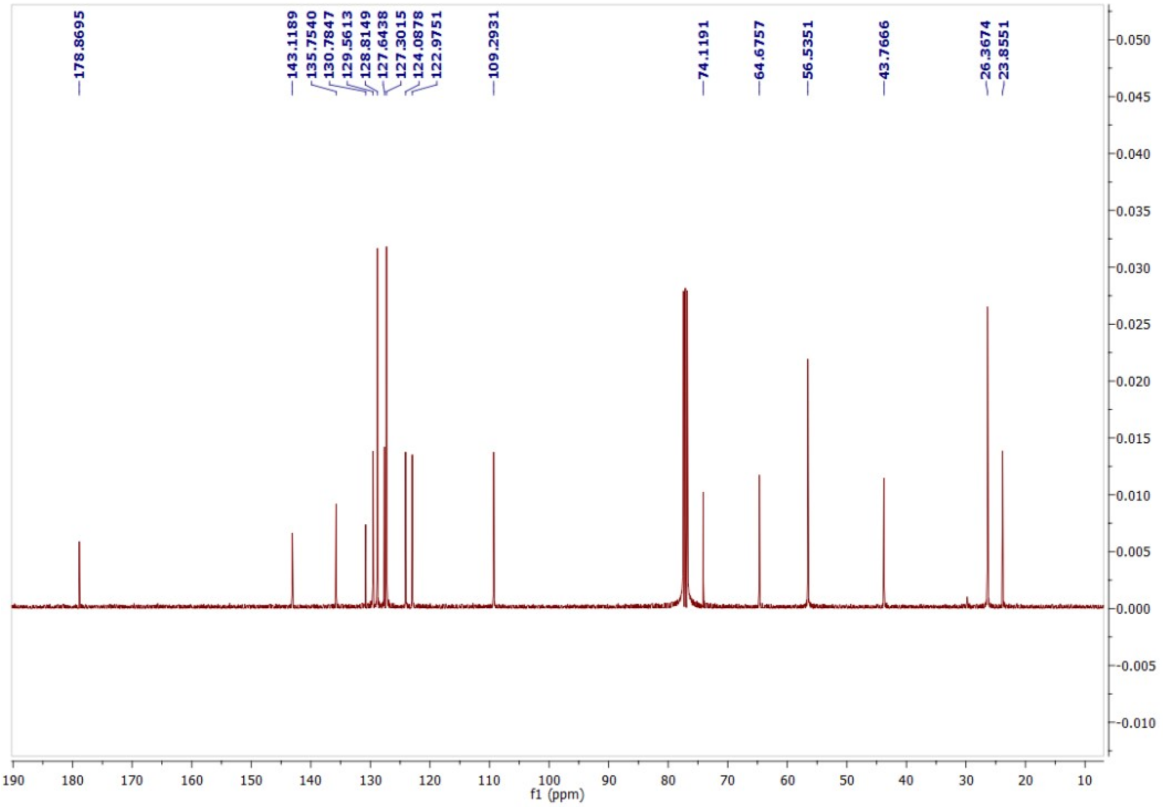

5. **^{1}H NMR** 1-Benzyl-3-hydroxy-3-(pyrrolidin-1-ylmethyl)indolin-2-one(**4c**)

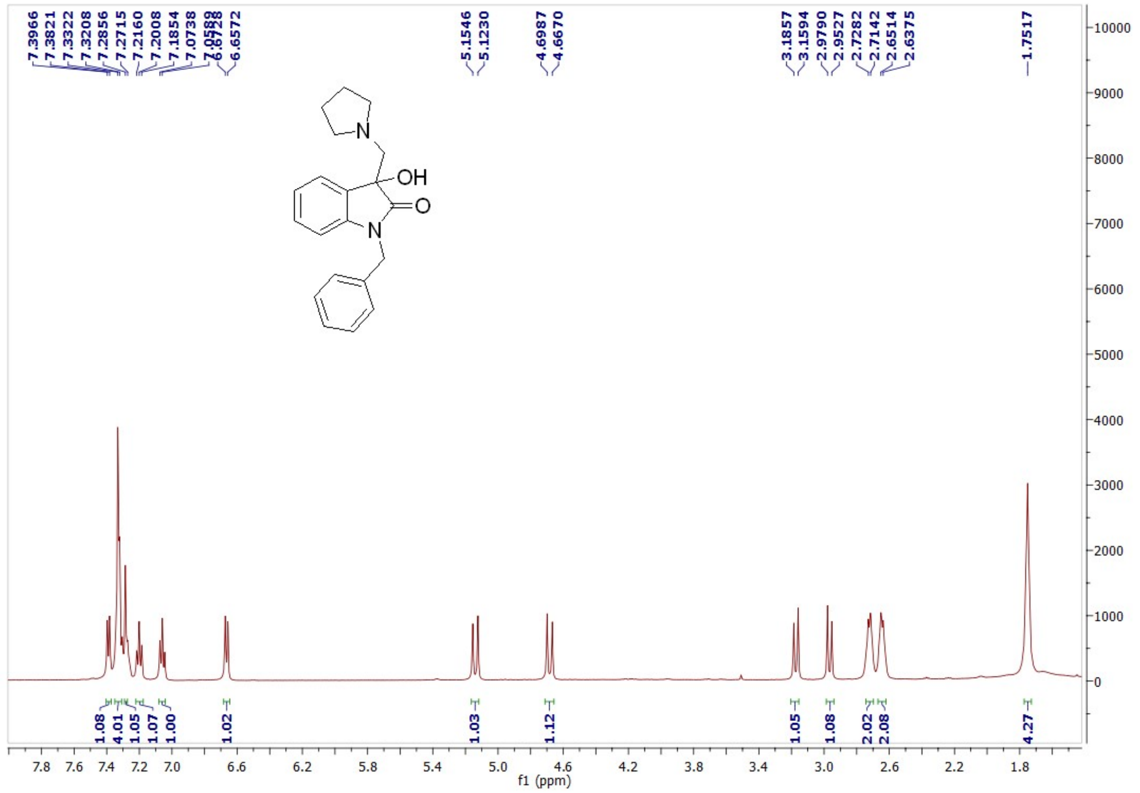

6. **^{13}C NMR** 1-Benzyl-3-hydroxy-3-(pyrrolidin-1-ylmethyl)indolin-2-one (**4c**)

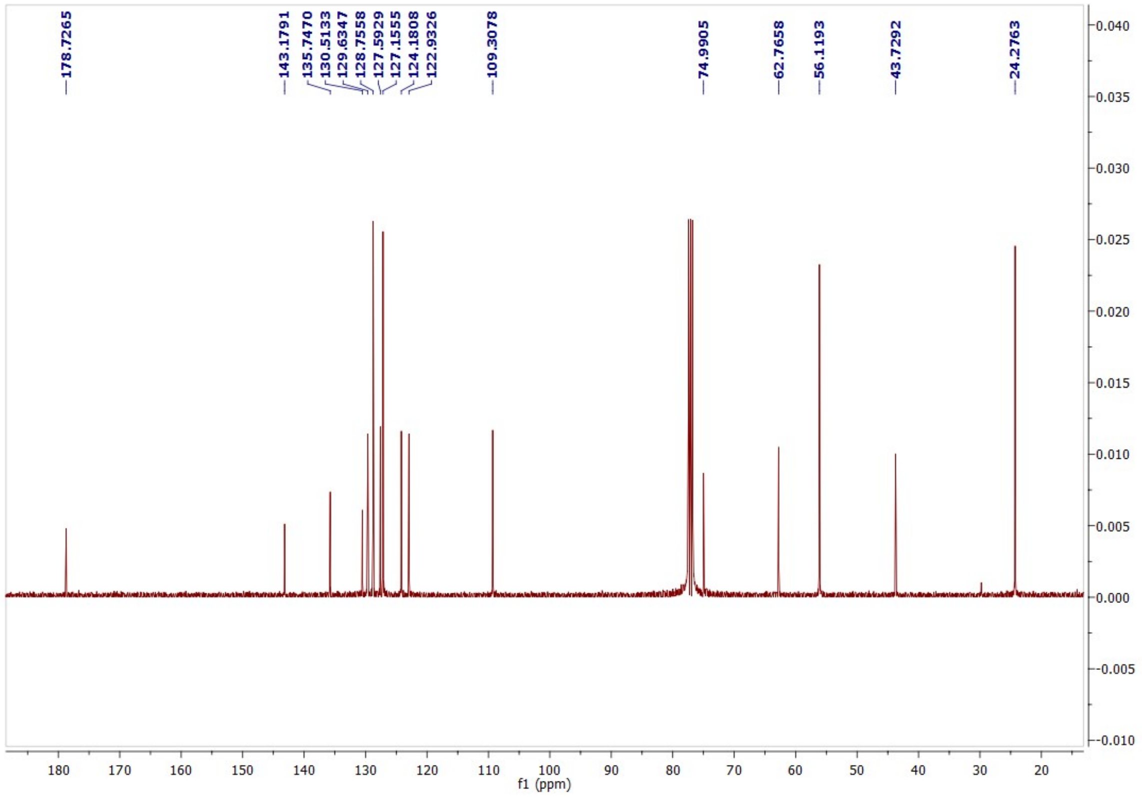

7. **^{1}H NMR** 1-Benzyl-5-bromo-3-hydroxy-3-(morpholinomethyl)indolin-2-one (**4d**)

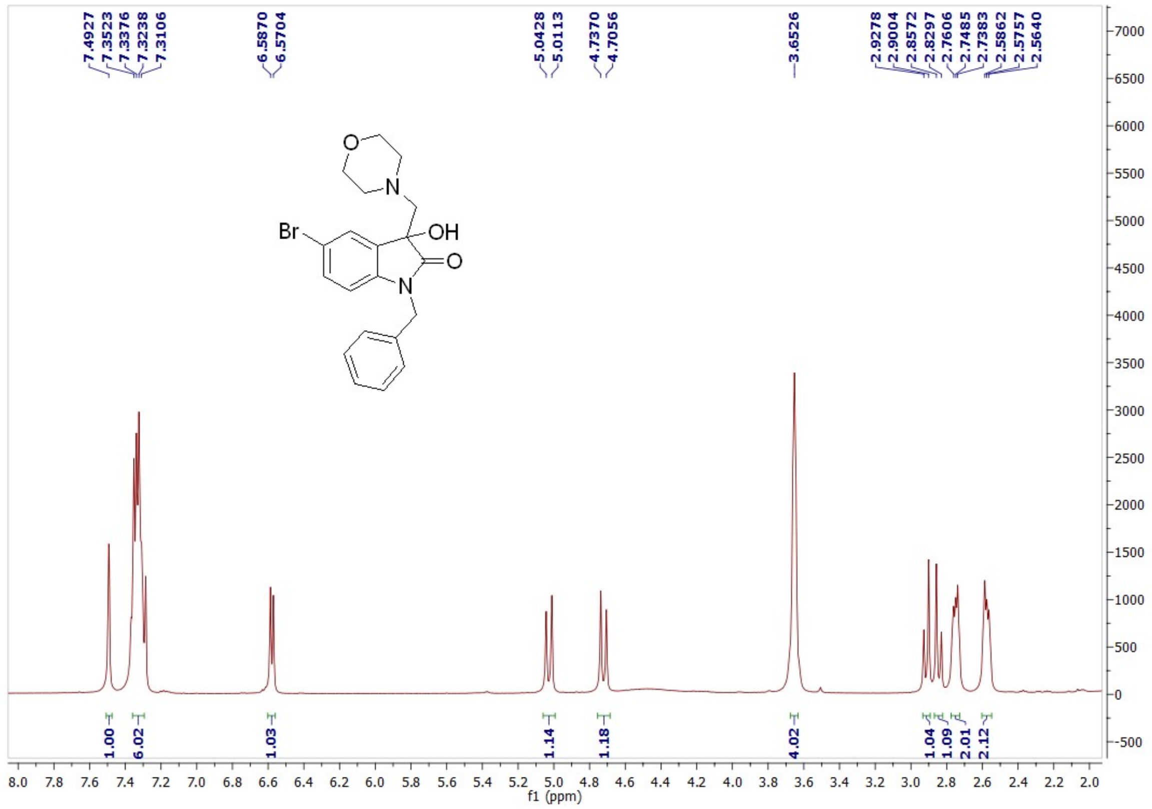

8. **^{13}C NMR** 1-Benzyl-5-bromo-3-hydroxy-3-(morpholinomethyl)indolin-2-one (**4d**)

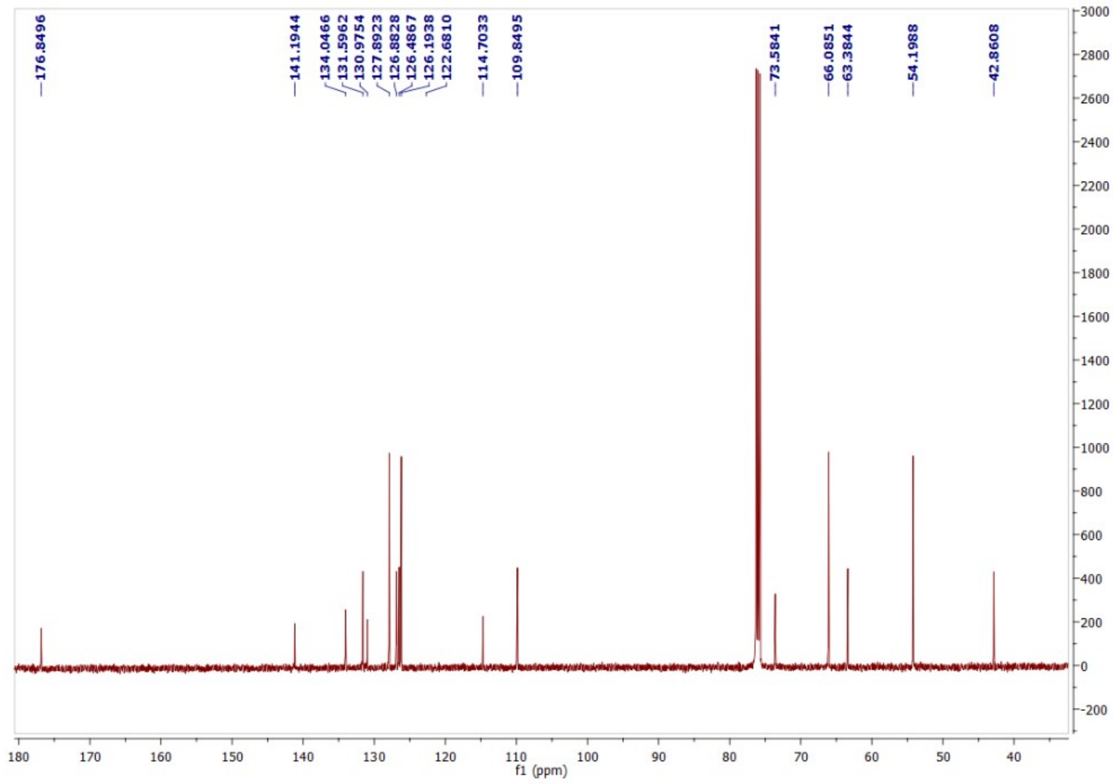

9. **^{1}H NMR** 1-Benzyl-5-bromo-3-hydroxy-3-(piperidin-1-ylmethyl)indolin-2-one (**4e**)

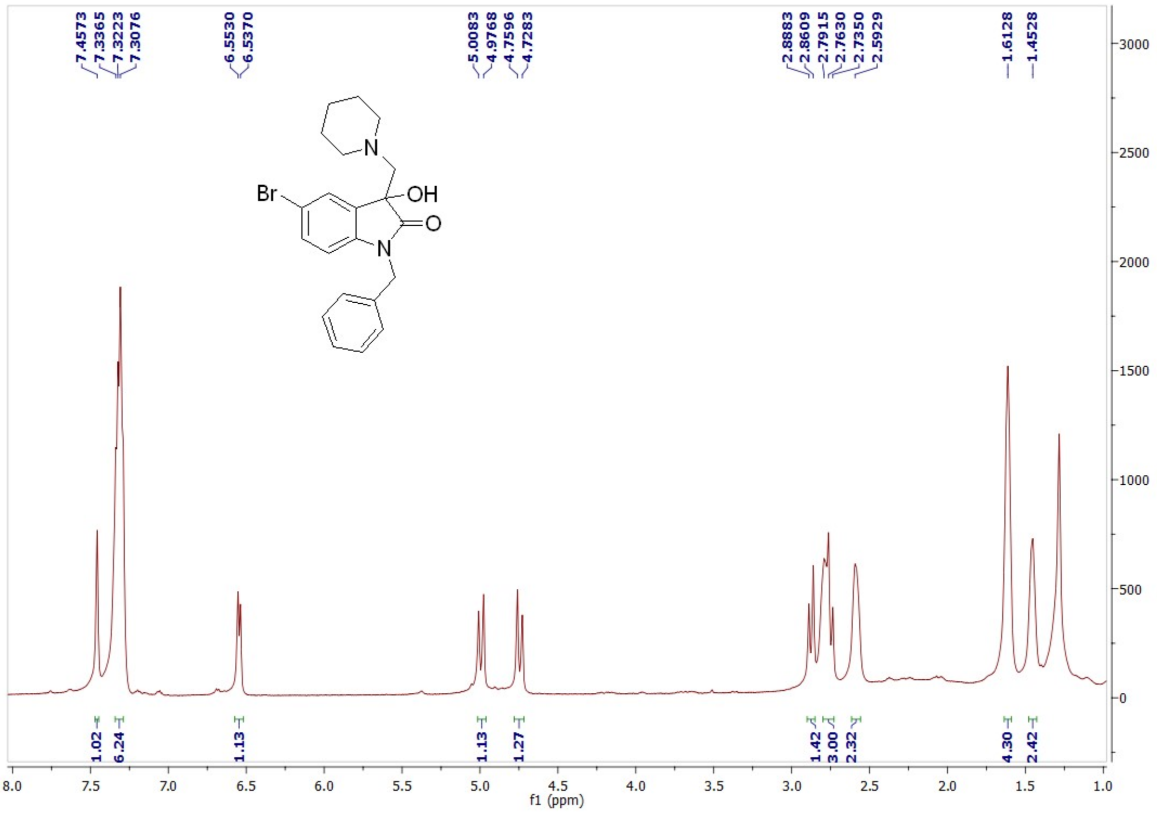

10. **^{13}C NMR** 1-Benzyl-5-bromo-3-hydroxy-3-(piperidin-1-ylmethyl)indolin-2-one (**4e**)

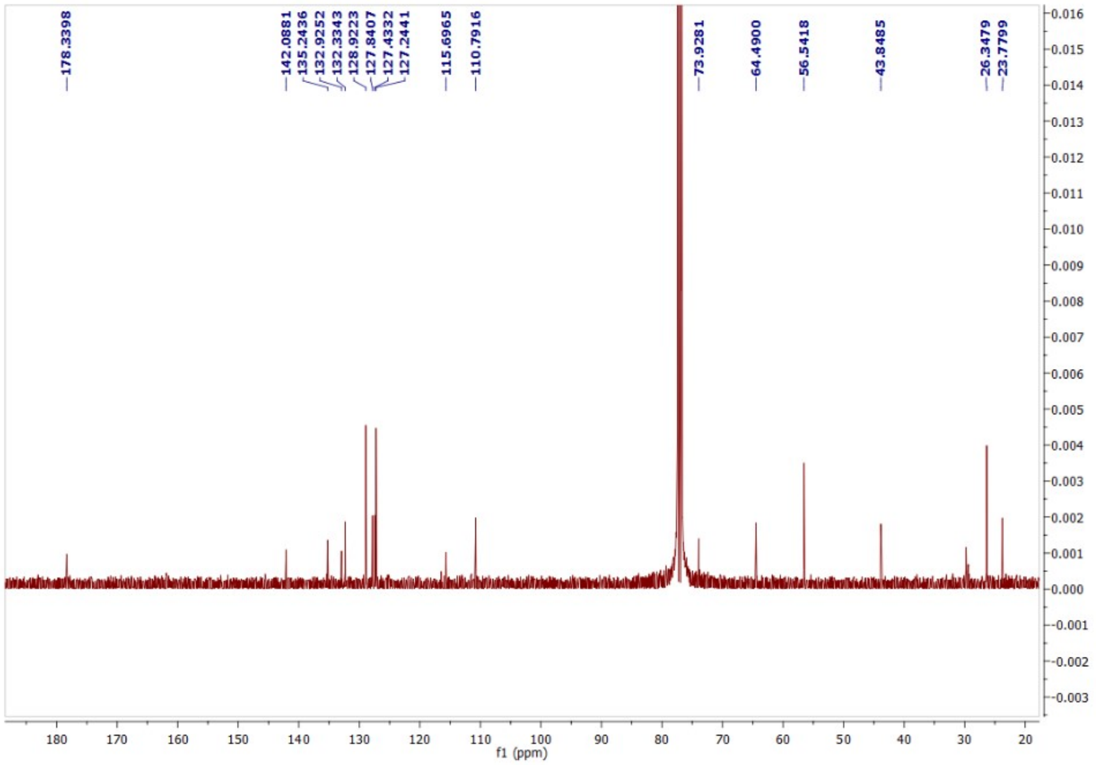

11. **^{1}H NMR** 1-Benzyl-5-bromo-3-hydroxy-3-(pyrrolidin-1-ylmethyl)indolin-2-one (**4f**)

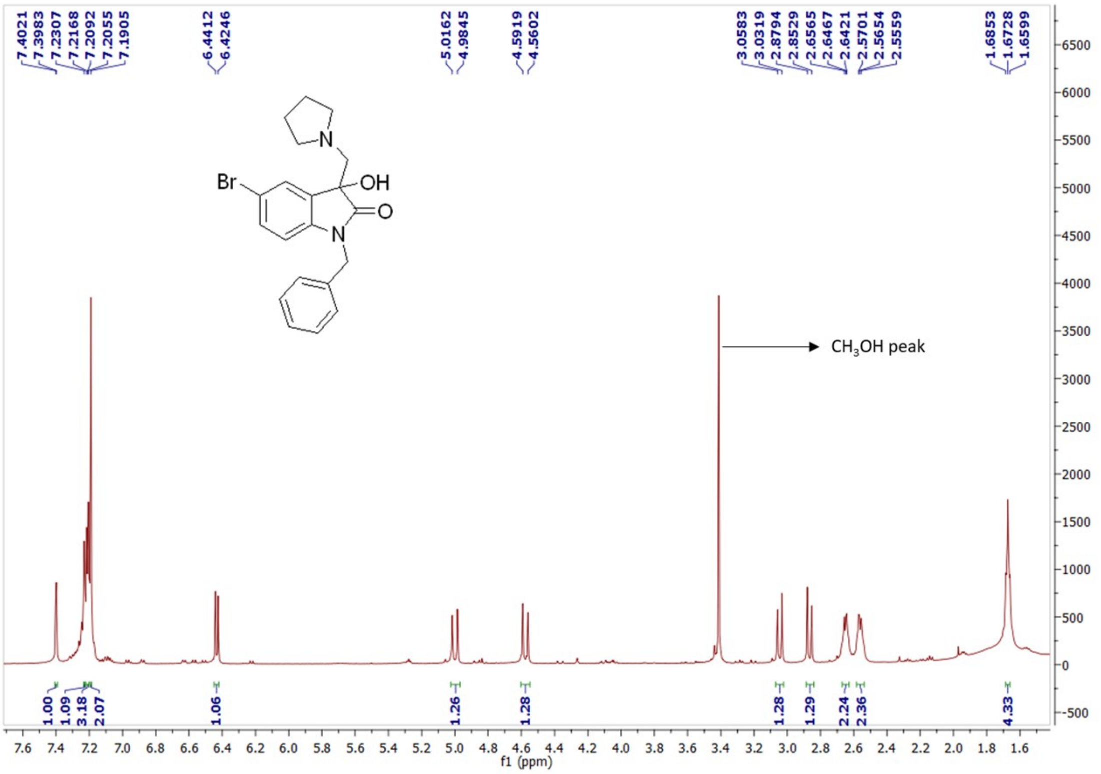

12. **^{13}C NMR** 1-Benzyl-5-bromo-3-hydroxy-3-(pyrrolidin-1-ylmethyl)indolin-2-one (**4f**)

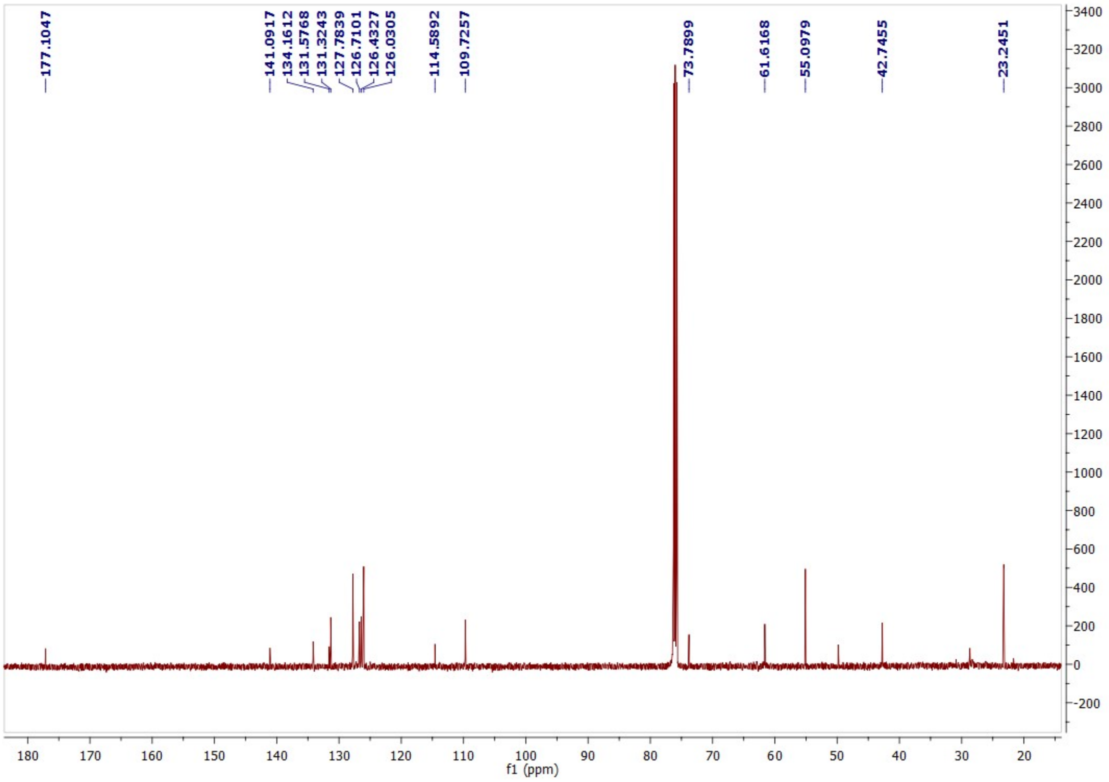

13. **^{1}H NMR** 1-Benzyl-5-chloro-3-hydroxy-3-(morpholinomethyl)indolin-2-one (**4g**)

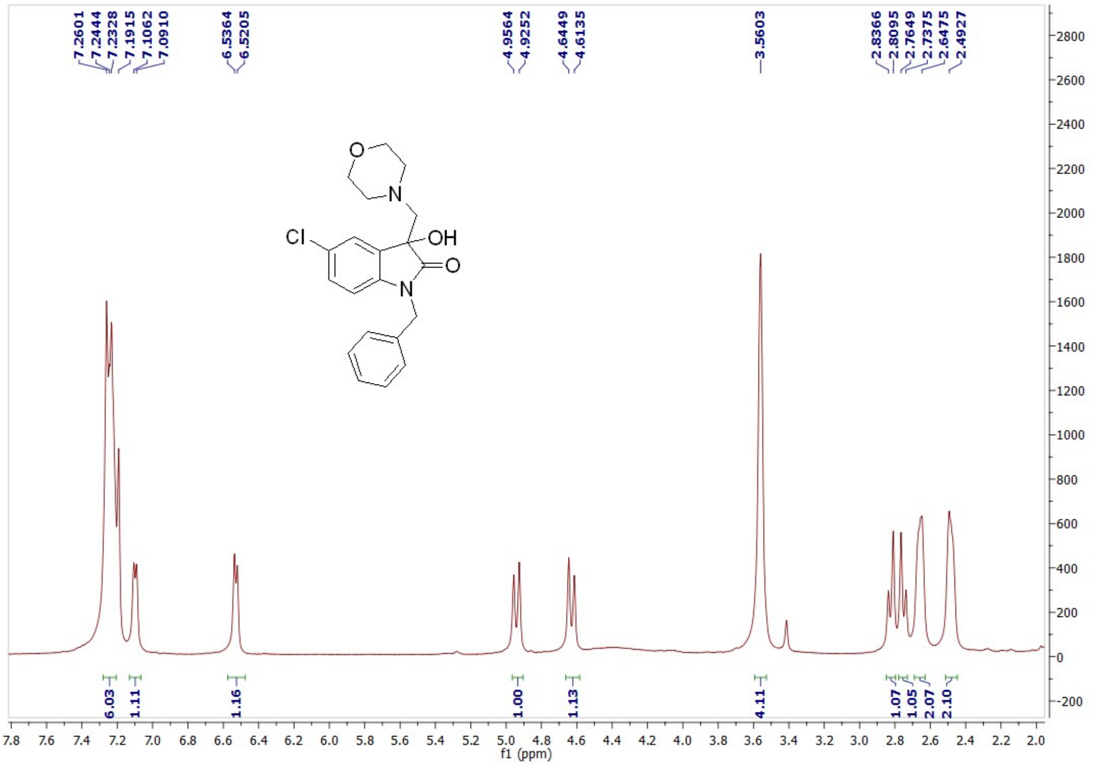

14. **^{13}C NMR** 1-Benzyl-5-chloro-3-hydroxy-3-(morpholinomethyl)indolin-2-one (**4g**)

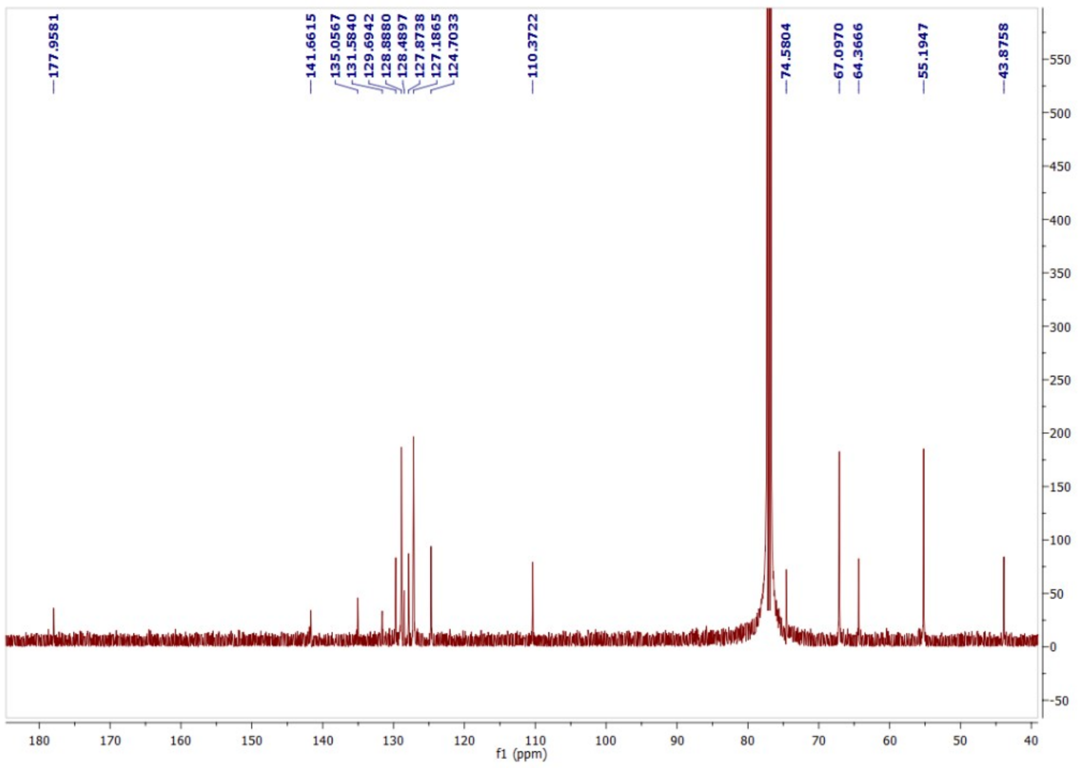

15. **^{1}H NMR** 1-Benzyl-5-chloro-3-hydroxy-3-(pyrrolidin-1-ylmethyl)indolin-2-one (**4i**)

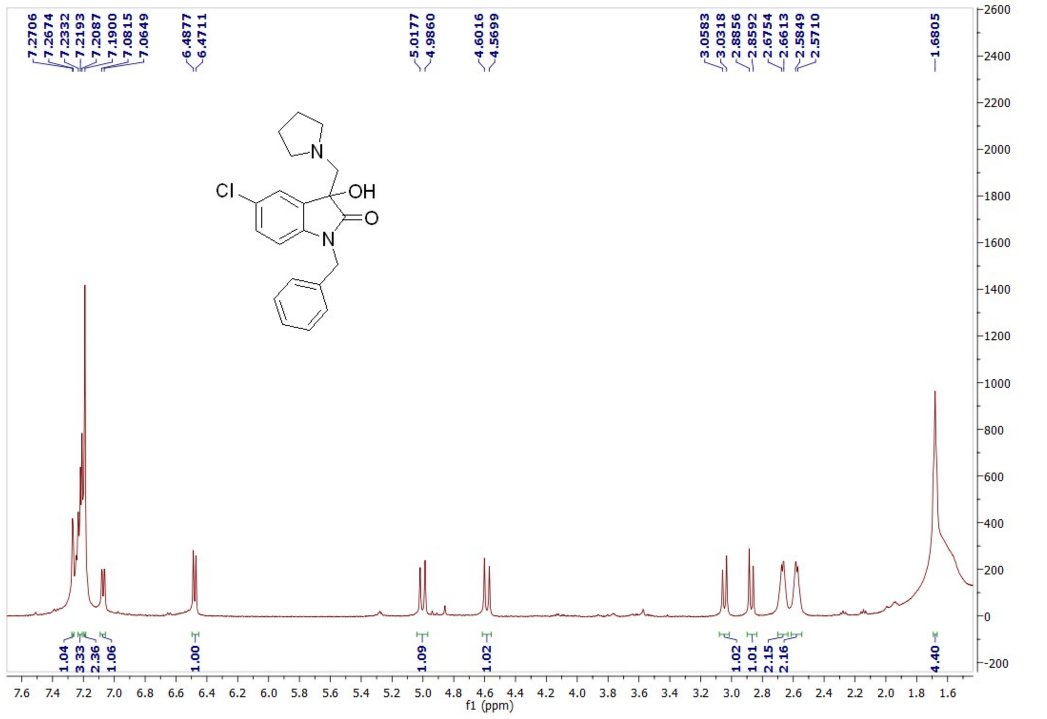

.

16. **^{13}C NMR** 1-Benzyl-5-chloro-3-hydroxy-3-(pyrrolidin-1-ylmethyl)indolin-2-one (**4i**)

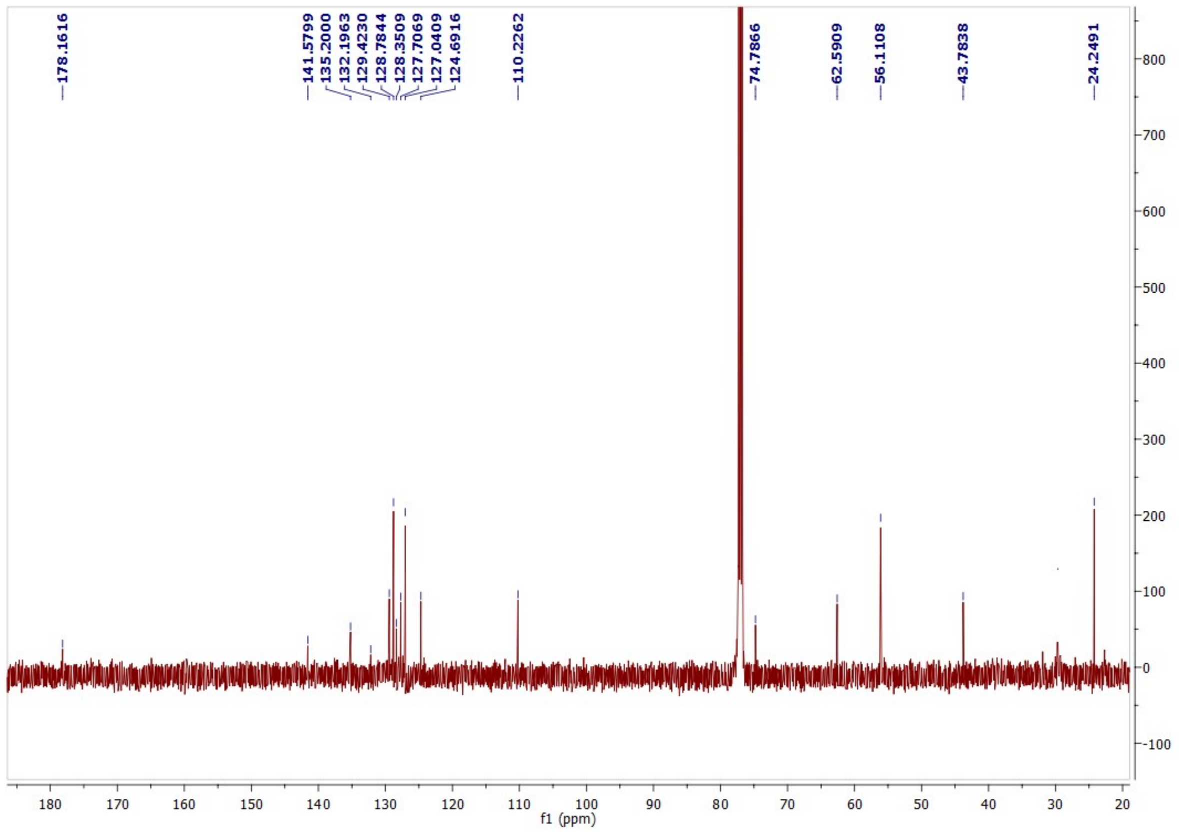

17. **^{1}H NMR** 1-Benzyl-5,7-dibromo-3-hydroxy-3-(morpholinomethyl)indolin-2-one (**4j**)

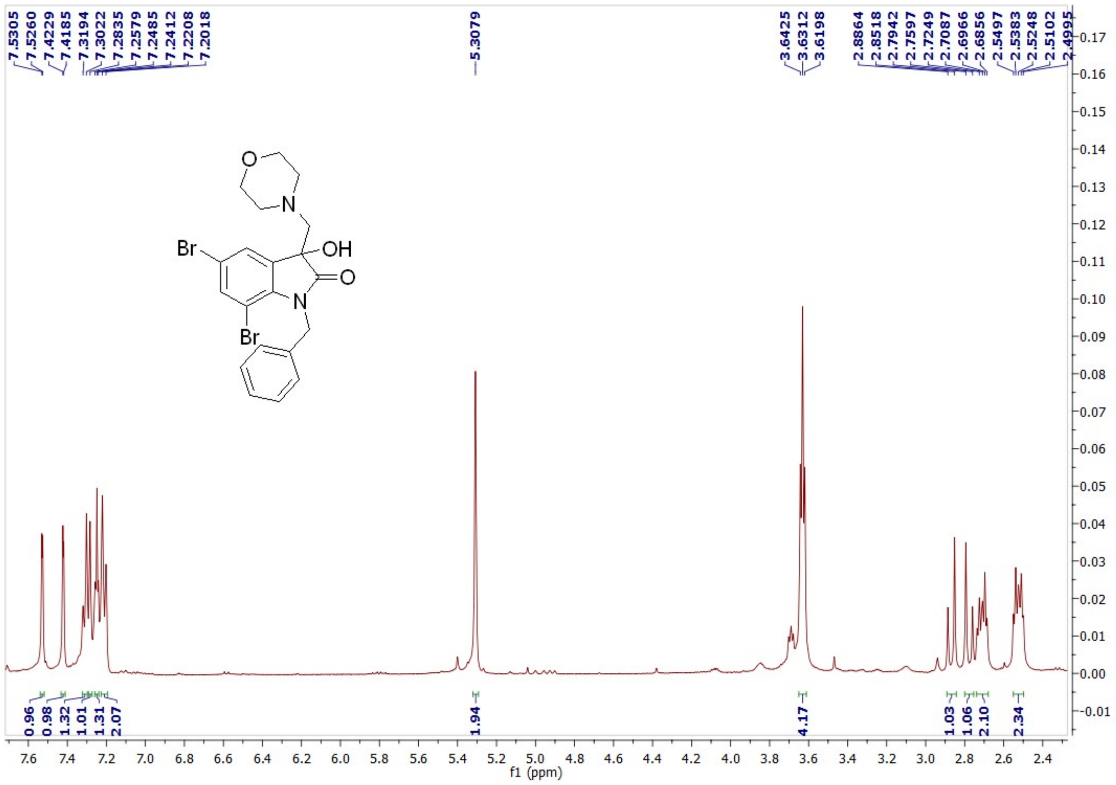

18. **^{13}C NMR** 1-Benzyl-5,7-dibromo-3-hydroxy-3-(morpholinomethyl)indolin-2-one (**4j**)

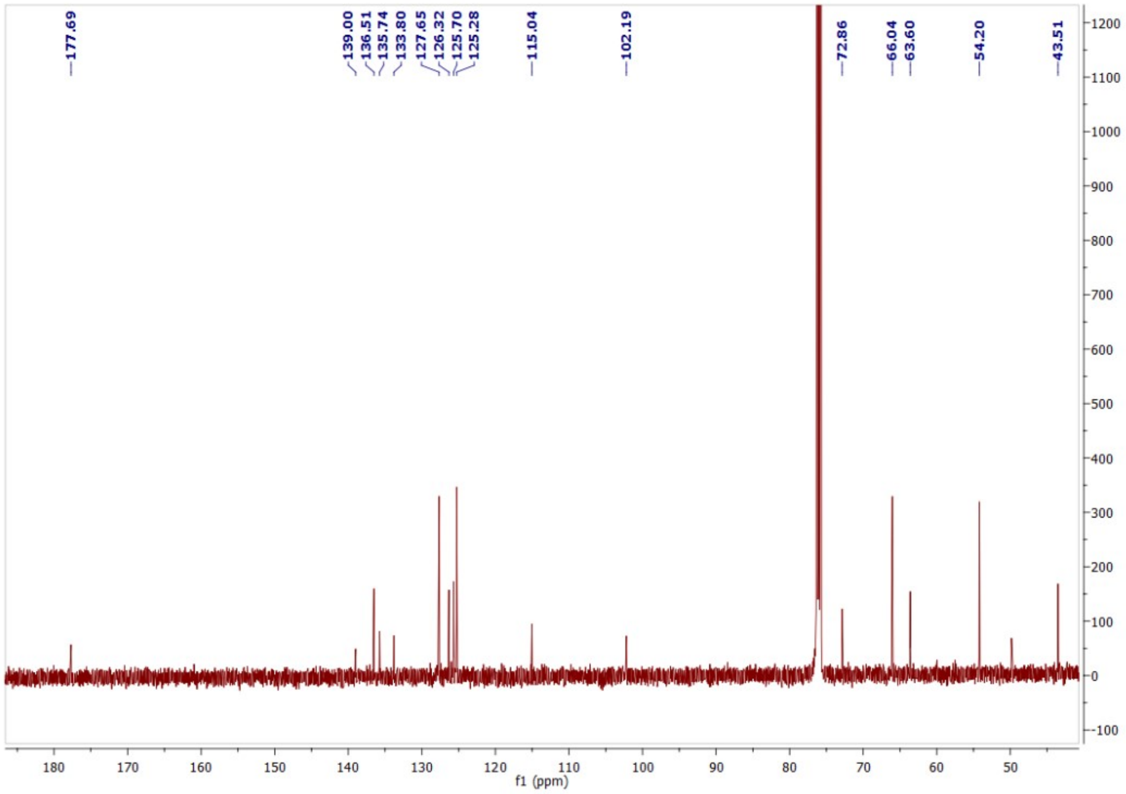

19. **^{1}H NMR** 1-Benzyl-5,7-dibromo-3-hydroxy-3-(piperidin-1-ylmethyl)indolin-2-one (**4k**)

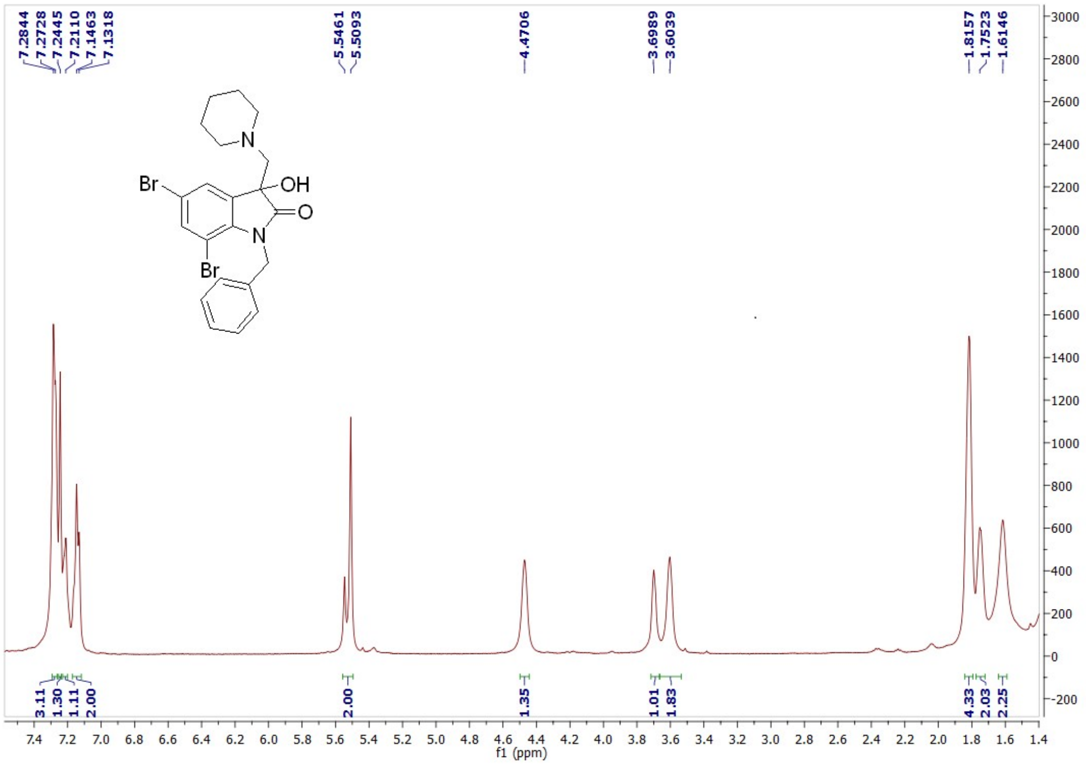

20. **^{13}C NMR** 1-Benzyl-5,7-dibromo-3-hydroxy-3-(piperidin-1-ylmethyl)indolin-2-one (**4k**)

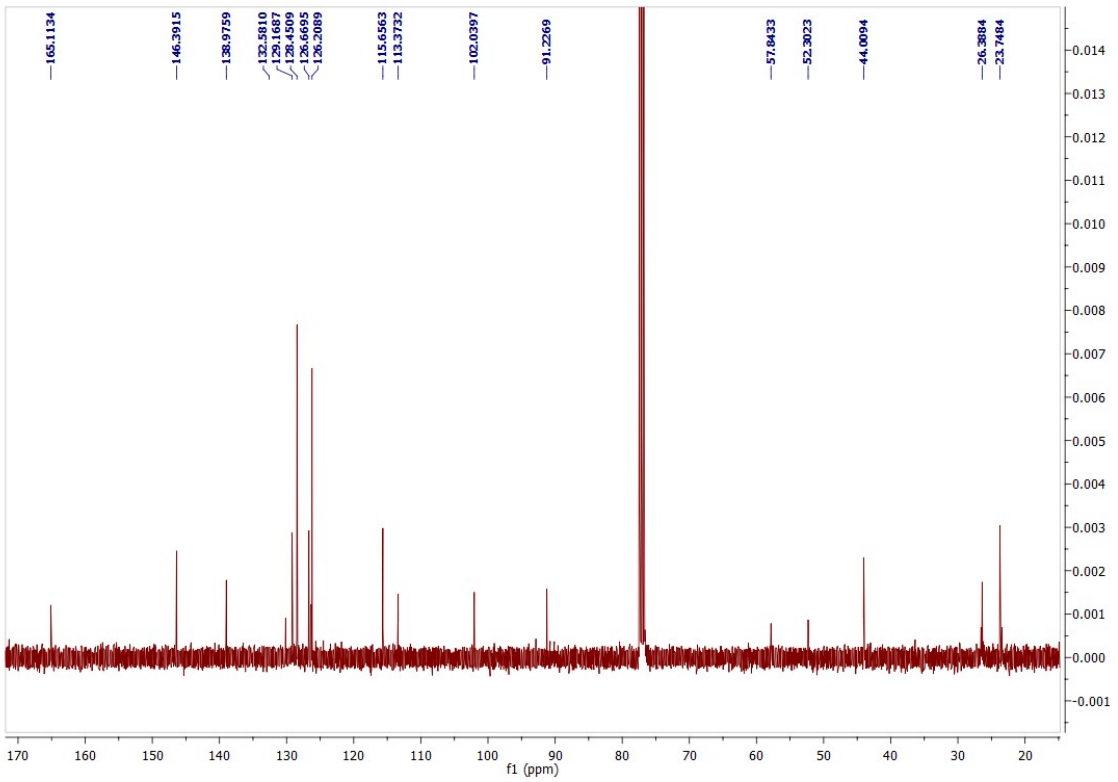

21. **^{1}H NMR** 3-Hydroxy-3-(morpholinomethyl)indolin-2-one (**4m**)

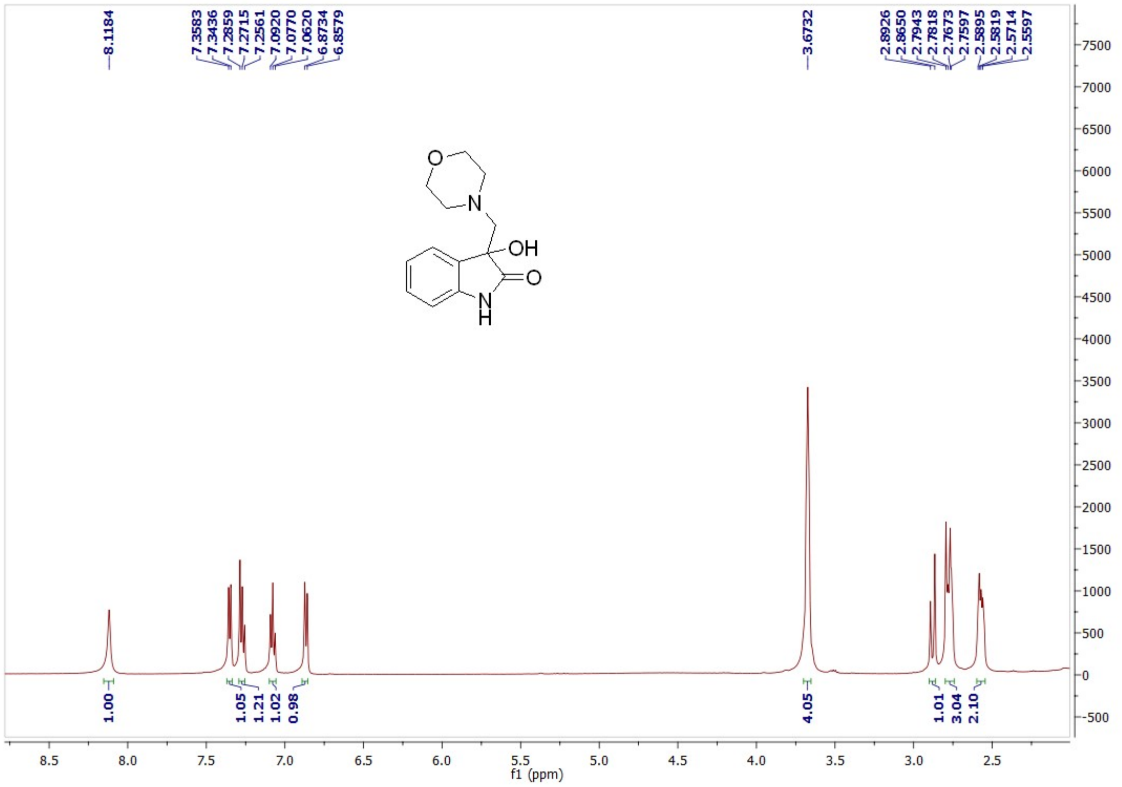

22. **^{13}C NMR** 3-Hydroxy-3-(morpholinomethyl)indolin-2-one (**4m**)

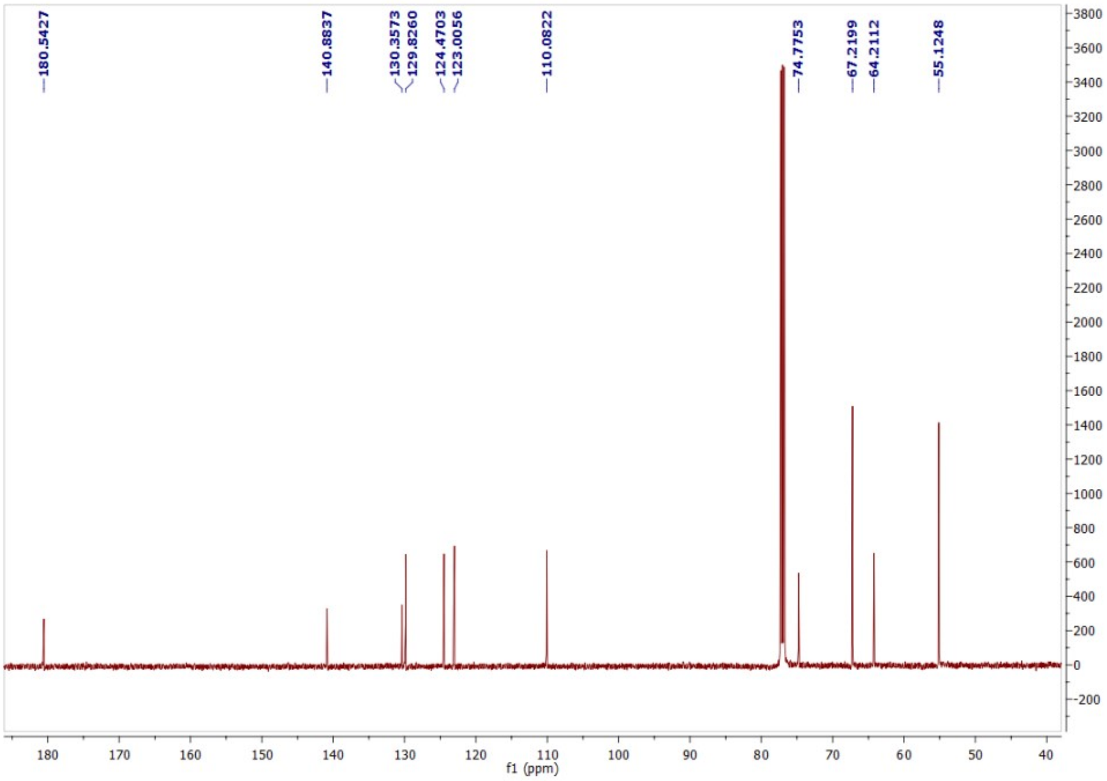

23. **^{1}H NMR** 3-Hydroxy-3-(piperidin-1-ylmethyl)indolin-2-one (**4n**)

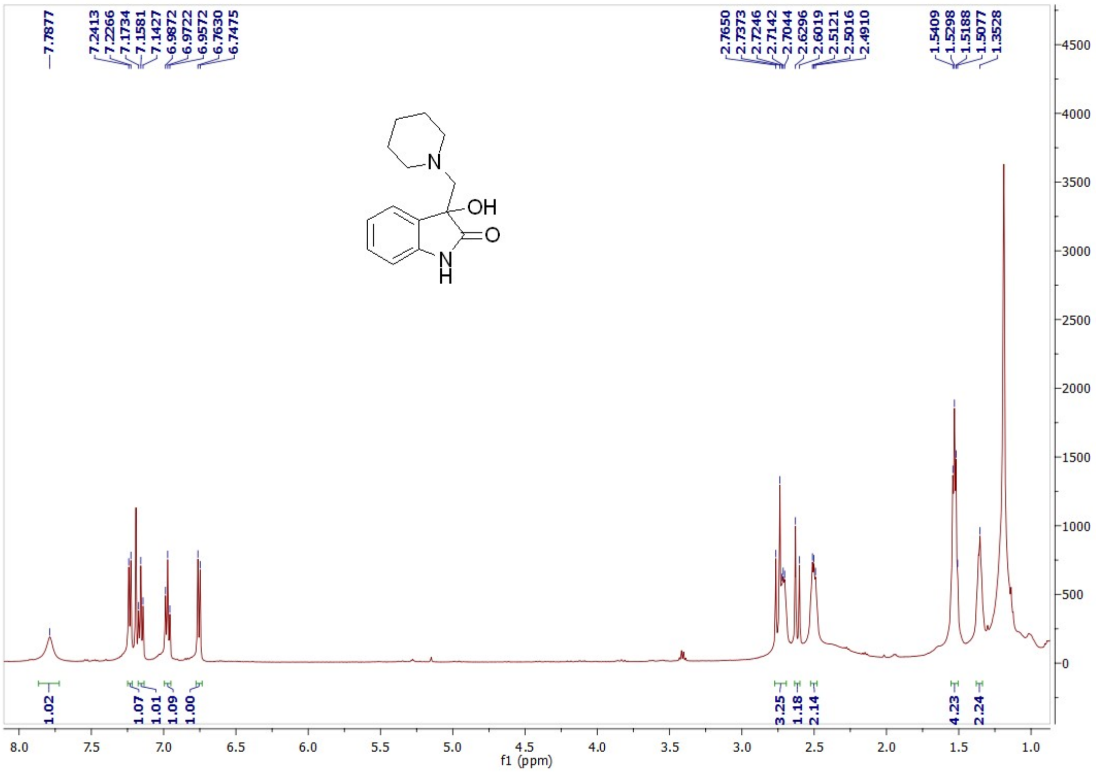

24. **^{13}C NMR** 3-Hydroxy-3-(piperidin-1-ylmethyl)indolin-2-one (**4n**)

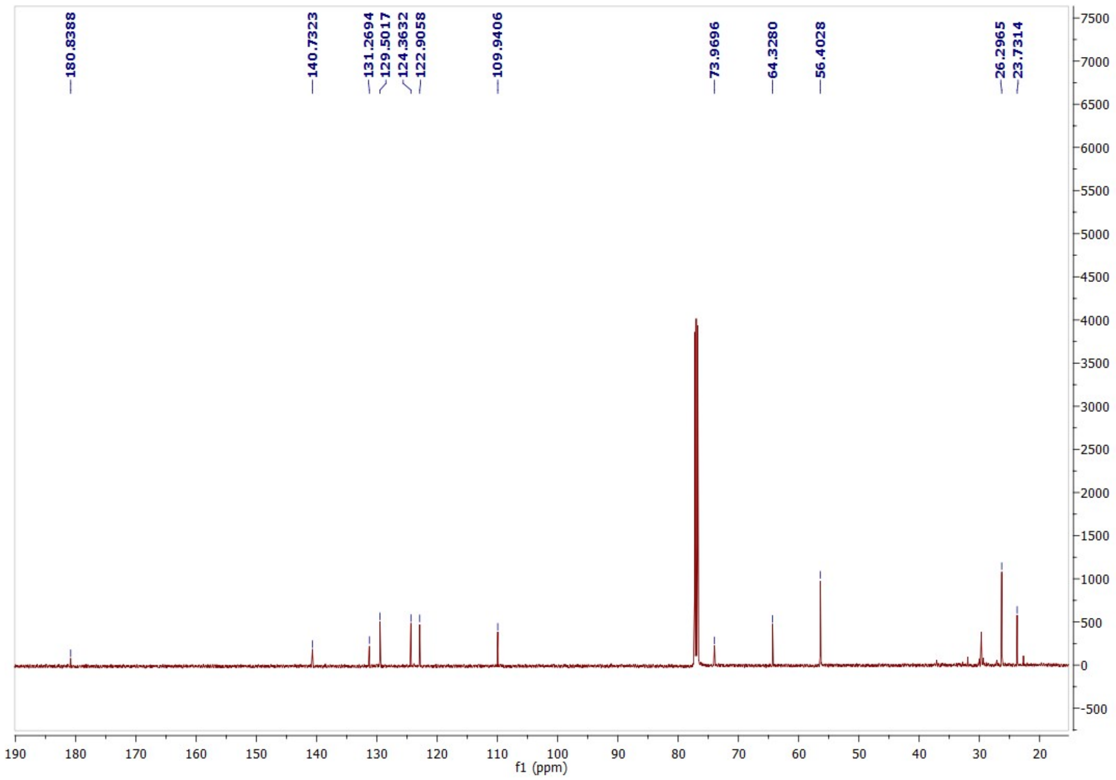

25. **^{1}H NMR** 3-Hydroxy-3-(pyrrolidin-1-ylmethyl)indolin-2-one (**4o**)

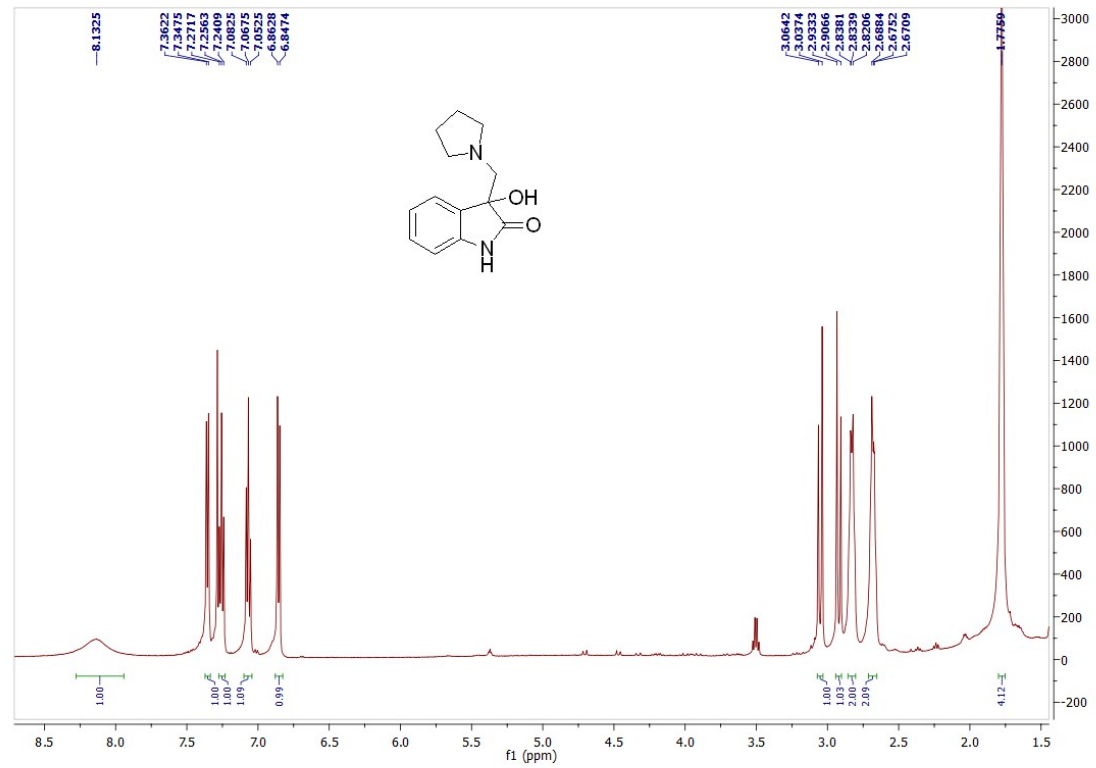

26. **^{13}C NMR** 3-ydroxy-3-(pyrrolidin-1-ylmethyl)indolin-2-one (**4o**)

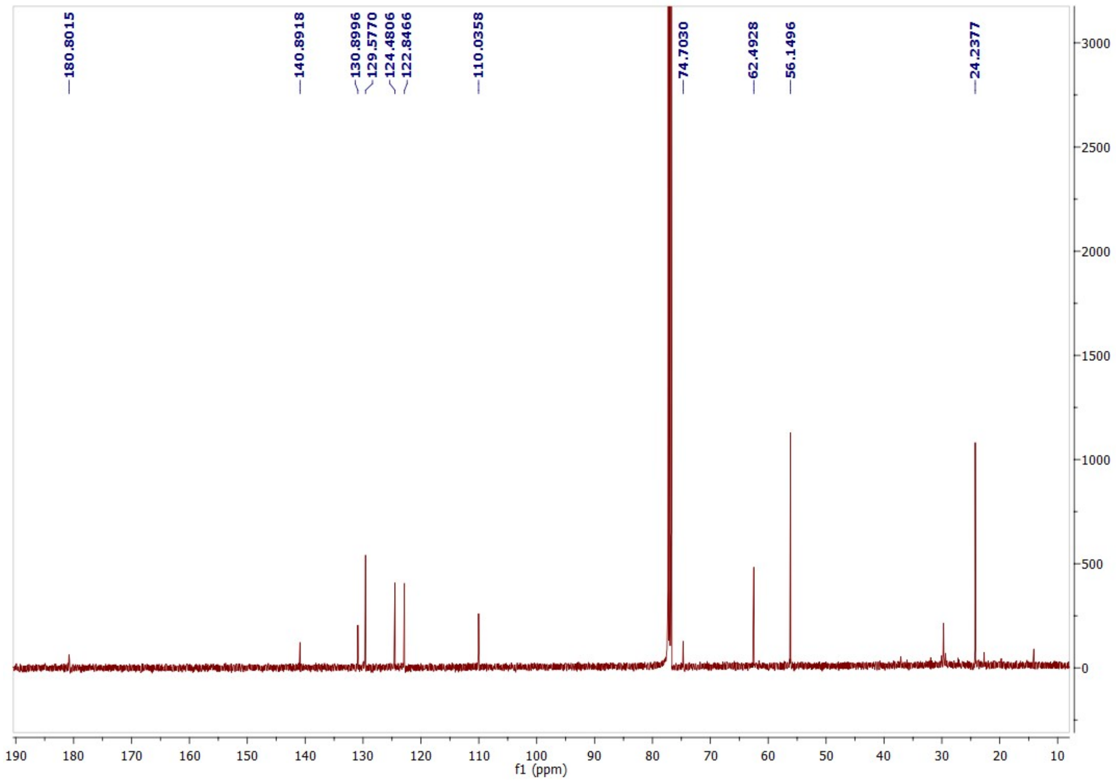

Supplement: RA-013-D3RA05869G-s001 [file RA-013-D3RA05869G-s001.pdf]
